# Mitochondrial and Cytoplasmic ROS Have Opposing Effects on Lifespan

**DOI:** 10.1371/journal.pgen.1004972

**Published:** 2015-02-11

**Authors:** Claire E. Schaar, Dylan J. Dues, Katie K. Spielbauer, Emily Machiela, Jason F. Cooper, Megan Senchuk, Siegfried Hekimi, Jeremy M. Van Raamsdonk

**Affiliations:** 1 Laboratory of Aging and Neurodegenerative Disease (LAND), Center for Neurodegenerative Science, Van Andel Research Institute, Grand Rapids, Michigan, United States of America; 2 Department of Translational Science and Molecular Medicine, Michigan State University, Grand Rapids, Michigan, United States of America; 3 Department of Genetics, Michigan State University, East Lansing, Michigan, United States of America; 4 Department of Biology, McGill University, Montreal, Quebec, Canada; Stanford University Medical Center, UNITED STATES

## Abstract

Reactive oxygen species (ROS) are highly reactive, oxygen-containing molecules that can cause molecular damage within the cell. While the accumulation of ROS-mediated damage is widely believed to be one of the main causes of aging, ROS also act in signaling pathways. Recent work has demonstrated that increasing levels of superoxide, one form of ROS, through treatment with paraquat, results in increased lifespan. Interestingly, treatment with paraquat robustly increases the already long lifespan of the *clk-1* mitochondrial mutant, but not other long-lived mitochondrial mutants such as *isp-1* or *nuo-6*. To genetically dissect the subcellular compartment in which elevated ROS act to increase lifespan, we deleted individual superoxide dismutase (*sod*) genes in *clk-1* mutants, which are sensitized to ROS. We find that only deletion of the primary mitochondrial sod gene, sod-2 results in increased lifespan in *clk-1* worms. In contrast, deletion of either of the two cytoplasmic *sod* genes, *sod-1* or *sod-5*, significantly decreases the lifespan of *clk-1* worms. Further, we show that increasing mitochondrial superoxide levels through deletion of *sod-2* or treatment with paraquat can still increase lifespan in *clk-1;sod-1* double mutants, which live shorter than *clk-1* worms. The fact that mitochondrial superoxide can increase lifespan in worms with a detrimental level of cytoplasmic superoxide demonstrates that ROS have a compartment specific effect on lifespan – elevated ROS in the mitochondria acts to increase lifespan, while elevated ROS in the cytoplasm decreases lifespan. This work also suggests that both ROS-dependent and ROS-independent mechanisms contribute to the longevity of *clk-1* worms.

## Author Summary

The accumulation of oxidative damage caused by reactive oxygen species (ROS) has been proposed to be one of the main causes of aging. However, recent work indicates that low levels of ROS can be beneficial and promote longevity. In this paper, we use a long-lived mitochondrial mutant *C*. *elegans* strain *clk-1* to further examine the relationship between ROS and lifespan. While it was originally believed that *clk-1* mutants had increased lifespan as a result of decreased ROS production, ROS levels have been shown to be increased in *clk-1* worms. We show that this increase in ROS is required for the longevity of *clk-1* worms as treatment with antioxidants decreases *clk-1* lifespan. Further, by using a genetic approach to increase ROS in specific subcellular compartments, we show that the location of the ROS is crucial in determining its effect on lifespan. Increasing ROS in the mitochondria markedly increases *clk-1* lifespan, while increasing ROS in the cytoplasm decreases it. Finally, we show that the effect of increased ROS on stress resistance and physiologic rates is also dependent on the location of ROS within the cell but that both of these factors can be experimentally dissociated from lifespan.

## Introduction

Reactive oxygen species (ROS) are toxic oxygen-containing molecules that can cause molecular damage within the cell. While it has been proposed that ROS-mediated damage contributes to aging [1], ROS have also been shown to act in signaling pathways [2]. In fact, recent work has demonstrated that mildly increasing ROS levels through treatment with the superoxide-generator paraquat can promote longevity [3–5]. In addition, elevated ROS have been proposed to alter physiologic rates [4,6,7] and contribute to the longevity of multiple long-lived mutants including *clk-1* worms [3,7,8].

The *clk-1* gene, which was identified in the worm *Caenorhabditis elegans*, was one of the first genes shown to increase lifespan in any organism [9,10]). *clk-1* encodes a hydroxylase involved in the synthesis of ubiquinone (coenzyme Q) [11], a redox-active lipid which acts as an electron carrier in the electron transport chain of the mitochondria [12]. Worms with a mutation in *clk-1* exhibit an overall slowing of numerous physiological rates including slow development, slow defecation cycle length, decreased brood size and slow rate of thrashing [9]. Interestingly, *clk-1* worms also exhibit a slow rate of aging leading to a significant extension of mean and maximum lifespan [10].

As *clk-1* worms have been shown to have a decreased rate of oxidative phosphorylation compared to wild-type worms [13–15], as well as decreased electron transport in the mitochondria [16,17], it was originally believed that the increased longevity of *clk-1* worms resulted from decreased production of ROS as a result of less electron transport chain activity. However, altered mitochondrial function in *clk-1* worms could also lead to increased leakage of electrons thereby increasing ROS generation. Distinguishing between these two possibilities has been made difficult by an inability to measure ROS directly in live worms [18].

Measurement of oxidative damage in *clk-1* worms has demonstrated that *clk-1* worms have decreased levels of protein carbonylation [14,19], decreased lipofuscin accumulation [20], and decreased 4-HNE [21,22]. In contrast, *clk-1* worms have increased levels of F3-isoprostanes [23]. Attempts to measure ROS levels using redox dyes have suggested that ROS levels are increased in *clk-1* worms. *clk-1* worms have increased 2’,7’-dichlorofluorescein diacetate (DCF) fluorescence in whole worm extracts and increased dihydroethidium (DHE) fluorescence in the heads of whole worms [5], as well as increased DCF fluorescence (overall ROS) but normal MitoSox staining (superoxide levels) in isolated mitochondria [3]. In addition, ROS production, as measured by Amplex Red, which detects hydrogen peroxide, was found to be normal in whole mitochondria but increased in sub-mitochondrial particles from *clk-1* worms [22]. Superoxide production potential has also been shown to be increased in *clk-1* worms using a lucigenin-mediated light production assay [20]. Combined, these results indicate that *clk-1* worms have increased levels of ROS but decreased levels of at least some types of oxidative damage. As oxidative damage is a balance between ROS production, ROS detoxification and repair, this suggests that antioxidant defense or damage repair may be increased in *clk-1* worms. While the level of catalase activity has been shown to be increased in *clk-1* worms [24,25], differing results have been obtained as to whether or not SOD protein or mRNA are increased [14,15,26,27].

In this paper, we determine the contribution of ROS to *clk-1* lifespan and physiologic rates. We find that elevated ROS in *clk-1* worms results in the upregulation of multiple classes of antioxidant genes during adulthood. We show that increased levels of ROS in the mitochondria further increase *clk-1* lifespan, while cytoplasmic ROS decrease it. We also observe compartment specific effects of ROS on stress resistance and physiologic rates that do not account for the effects on lifespan. Finally, our data suggest that both ROS-dependent and ROS-independent mechanisms contribute to the longevity of *clk-1* worms.

## Results

### *clk-1* worms have increased antioxidant defenses

As *clk-1* worms have increased levels of ROS [3,5] but decreased oxidative damage [14,21], we measured the expression levels of antioxidant enzymes to determine the extent to which increased antioxidant defenses could account for the observed levels of oxidative damage in *clk-1* worms. We used quantitative real-time RT-PCR to measure the levels of four different antioxidant enzymes: superoxide dismutase (SOD), catalase (CTL), peroxiredoxin (PRDX) and thioredoxin (TRX). SOD detoxifies superoxide, CTL and PRDX detoxify hydrogen peroxide, and TRX reduces various substrates in the cell including PRDX. By examining all of the SODs, CTLs, PRDXs and TRXs, we were able to also determine which specific subcellular compartments had increased antioxidant defense.

**Figure 1:**
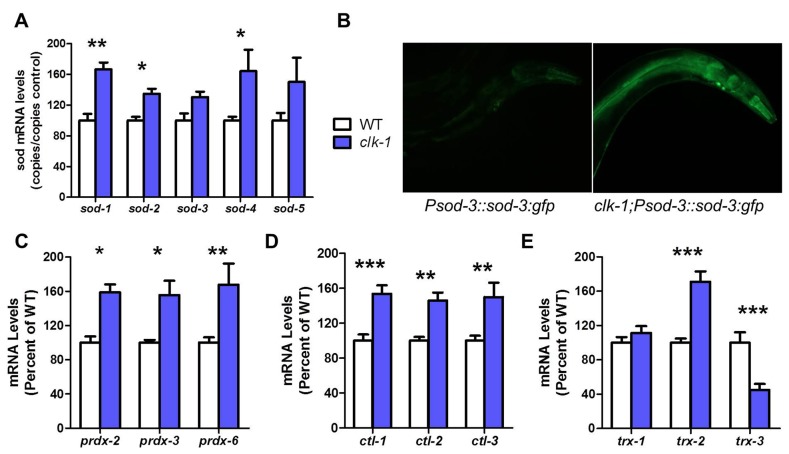
*clk-1* worms have increased antioxidant defenses. The expression of antioxidant genes was examined in day 1 adult worms by quantitative real-time RT-PCR. Compared to WT worms (white), *clk-1* worms (blue) showed increased expression of four different types of antioxidant genes including: (**A**) superoxide dismutases (*sod*), (**C**) peroxiredoxins (*prdx*), (**D**) catalases (*ctl*) and (**E**) thioredoxins (*trx*). The expression of SOD-3 protein is also significantly increased in *clk-1* worms as indicated by an increase in GFP intensity in *clk-1* worms expressing a SOD-3:GFP transgene under the *sod-3* gene promoter (**B**). Error bars indicate SEM. * p<0.05, ** p<0.01, *** p<0.001.

We found that all five *sod* genes are upregulated in *clk-1* worms compared to WT (Fig. 1A). We also observed increased expression of SOD-3 protein in *clk-1* worms expressing SOD-3:GFP under a *sod-3* promoter (Fig. 1B). Similarly, we observed increased expression of all three *prdx* genes (*prdx-2*, *prdx-3*, *prdx-6*), all three *ctl* genes (*ctl-1*, *ctl-2*, *ctl-3*) and *trx-2* in *clk-1* worms (Fig. 1C-E). In contrast, the levels of *trx-3*, which is expressed in the cytoplasm of the intestine [28], were significantly decreased in *clk-1* worms. Thus, *clk-1* worms exhibit significantly increased expression of antioxidant genes in multiple sub-cellular compartments of the cell including the cytoplasm (*sod-1*, *ctl-1*, *prdx-2*, *trx-1*), mitochondria (*sod-2*, *sod-3*, *trx-2*), peroxisome (*ctl-2*) and extra-cellularly (*sod-4*).

### *clk-1* worms are sensitive to acute oxidative stress but resistant to chronic oxidative stress in adulthood

**Figure 2:**
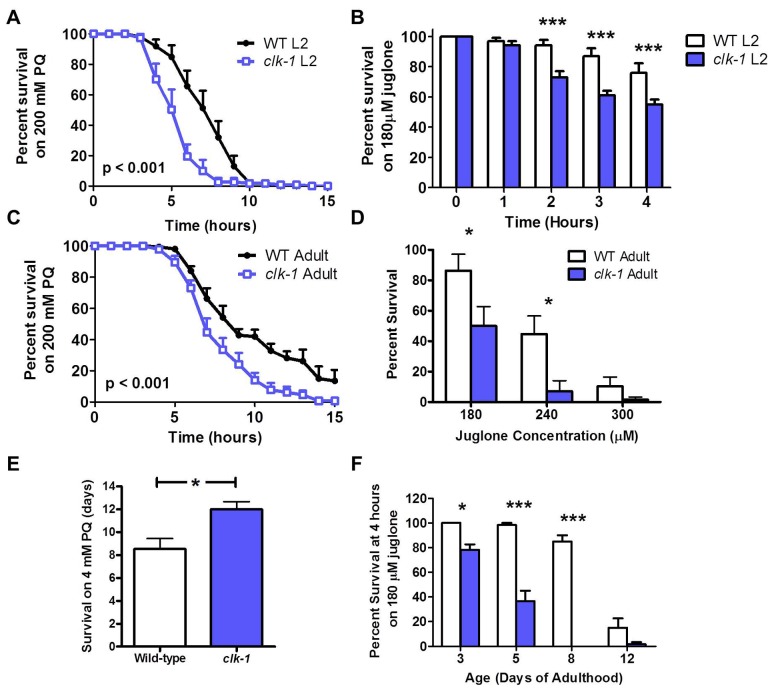
*clk-1* worms are sensitive to acute exposure to oxidative stress but resistant to chronic exposure. Sensitivity to oxidative stress was assessed during development and adulthood using two superoxide-generating compounds: paraquat (PQ) and juglone. Chronic assays of oxidative stress could only be performed with paraquat because the toxicity of juglone is decreased within 8 hours. During development, *clk-1* L2 larvae have decreased survival compared to wild-type worms under conditions of oxidative stress: **A**. 200 mM paraquat or **B**. 180 μM juglone. Similarly, in acute assays of oxidative stress assay on day 1 of adulthood, *clk-1* worms show decreased survival compared to wild-type worms after exposure to either **C**. 200 mM paraquat or **D**. different concentrations of juglone (180–300 μM), indicating increased sensitivity to oxidative stress. **E**. In a chronic oxidative stress assay where worms are exposed to 4 mM paraquat beginning on day 1 of adulthood after development on NGM plates, *clk-1* worms survive significantly longer than wild-type worms. **F**. However, *clk-1* worms remain sensitive to acute oxidative stress throughout adulthood when exposed to 180 μM juglone. Overall, this shows that *clk-1* worms are sensitive to acute oxidative stress throughout development and adulthood but are resistant to chronic oxidative stress during adulthood. Error bars indicate SEM. * p<0.05, *** p<0.001.

Having shown that *clk-1* worms have increased antioxidant defenses, we next wanted to determine whether this increase in antioxidant defense was sufficient to protect *clk-1* worms from their elevated ROS production. To do this, we measured sensitivity to oxidative stress during development and adulthood using two different superoxide-generating compounds, paraquat and juglone. At the L2 stage of development, we found that *clk-1* worms have decreased survival compared to WT on plates containing either 200 mM paraquat or 180 μM juglone (Fig. 2A,B). Similarly, we found that *clk-1* worms fail to develop to adulthood under conditions of oxidative stress that are insufficient to prevent the development of WT worms (S1A Fig.). Combined, this indicates that *clk-1* worms have increased sensitivity to oxidative stress during development.

To assess sensitivity to an acute exposure to oxidative stress during adulthood, we exposed *clk-1* and WT worms to plates containing 200 mM paraquat or 180–300 μM juglone. In both cases, *clk-1* worms had decreased survival compared to WT worms, indicating increased sensitivity to acute oxidative stress on day 1 of adulthood (Fig. 2C,D). In contrast, we found that in a chronic oxidative stress assay where worms were exposed to 4 mM paraquat beginning at day 1 of adulthood, *clk-1* worms show increased survival compared to WT worms (Fig. 2E). This suggests that either *clk-1* worms become resistant to oxidative stress during adulthood or that *clk-1* worms respond differently to acute versus chronic stresses. To distinguish between these two possibilities, we examined sensitivity to an acute exposure to oxidative stress at various time points throughout adulthood by treating worms with 180 μM juglone. We found that at each time point, *clk-1* worms exhibited increased sensitivity to oxidative stress compared to wild-type worms (Fig. 2F, S2 Fig.). Thus, *clk-1* worms are sensitive to acute exposure to oxidative stress but resistant to chronic exposure. The increased resistance to chronic oxidative stress is consistent with *clk-1* worms having decreased levels of oxidative damage. Note that testing sensitivity to chronic oxidative stress using juglone is unfeasible as its toxicity decreases rapidly within 8 hours of pouring plates (S1B Fig.).

### Stress response pathways are upregulated in *clk-1* worms and decline with age

As *clk-1* worms have been shown to have elevated levels of ROS, it is plausible that the elevated levels of ROS trigger a protective response that results in their increased resistance to chronic oxidative stress and decreased oxidative damage. To investigate the mechanism underlying the increased resistance to chronic oxidative stress in *clk-1* worms, we used reporter strains to monitor different stress response pathways that have been implicated in *clk-1* lifespan. We used a *gst-4* reporter to monitor upregulation of phase II detoxification pathways (*skn-1* target)[29], an *hsp-6* reporter to monitor the upregulation of the mitochondrial unfolded protein response (UPR)[30] and an *nhr-57* reporter to monitor the upregulation of a hypoxic response (*hif-1* target)[31].

**Figure 3:**
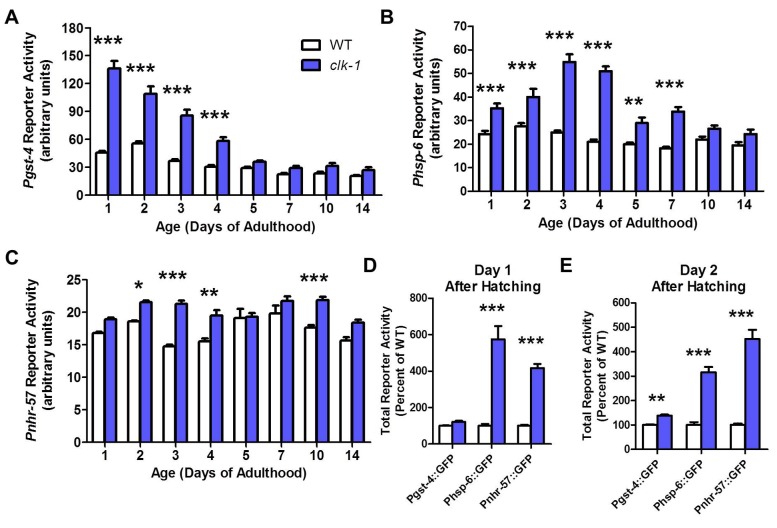
Stress responsive pathways in *clk-1* worms are upregulated during larval development and decline with age. *Pgst-4*, *Phsp-6* and *Pnhr-57* reporter constructs were used to monitor the upregulation of oxidative stress response, mitochondrial unfolded protein response (mitoUPR) and hypoxia response during development and aging in *clk-1* worms. All three pathways were increased on day 1 of adulthood. **A**. The upregulation of the oxidative stress response decreases with age. **B**. The increase in mitoUPR in *clk-1* worms continues to increase until day 3 of adulthood and then decreases with age. **C**. In contrast, the mild upregulation of the hypoxia response is relatively constant with increasing age. **D**. On day 1 after hatching the mitoUPR and hypoxia response are already activated in *clk-1* worm. **E**. On day 2 after hatching, all three stress response pathways are upregulated in *clk-1* worms. Error bars indicate SEM. * p<0.05, ** p<0.01, *** p<0.001.

On a wild-type background, *Pgst-4* reporter activity decreased mildly with increasing age (Fig. 3A). In *clk-1* worms, we found that activity of the *Pgst-4* reporter was already markedly increased on day 1 of adulthood (Fig. 3A). As in WT worms, *Pgst-4* reporter activity declined with age and was similar to WT worms at day 5 and beyond. Using the *Phsp-6* reporter, we found that the mitoUPR was upregulated in *clk-1* worms at day 1 of adulthood (Fig. 3B). In contrast to the *Pgst-4* reporter, the maximum increase was observed at days 3 and 4 of adulthood and then declined with age. Finally, with the *Pnhr-57* reporter, we observed a mild activation of a hypoxic response that was relatively constant as the worms aged (Fig. 3C).

To determine whether the decline in *Pgst-4* reporter activity resulted from a decrease in oxidative stress or a decrease in the ability to activate *skn-1* in response to oxidative stress, we treated day 1 adult worms and day 10 adult worms with 2 mM paraquat. We found that aged worms failed to activate the *gst-4* promoter (S3 Fig.). This suggests that the decline in *Pgst-4* reporter activity in *clk-1* worms results from a decrease in the ability to respond to oxidative stress with increasing age and does not indicate that the levels of oxidative stress decline to wild-type levels in *clk-1* worms. Interestingly, treatment with paraquat does not further increase *Pgst-4* reporter activity in *clk-1* worms suggesting that these worms already have a maximal response (S3 Fig.).

Since all of the reporters were already upregulated in young adult *clk-1* worms, we next sought to determine if there is a critical time period during development when these reporters become activated. Examination of *clk-1* and WT worms revealed that the *Phsp-6* and *Pnhr-57* reporters were already upregulated on day 1 after hatching (Fig. 3D), while the *Pgst-4* reporter was not significantly activated until day 2 (Fig. 3E). Combined this indicates that all three stress response pathways are engaged during embryonic or very early development and continue to be upregulated into adulthood.

### Antioxidant genes become upregulated during adulthood in *clk-1* worms

**Figure 4:**
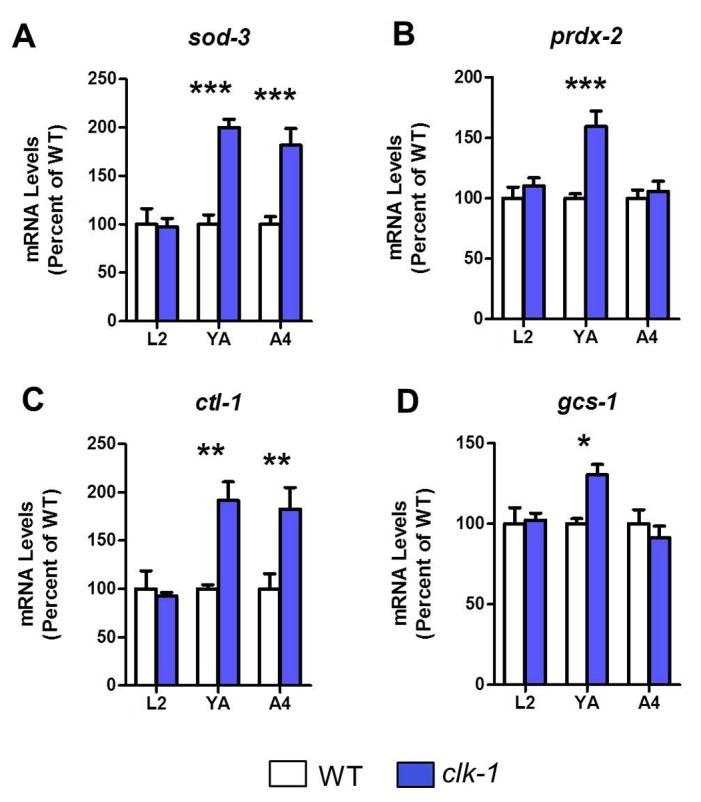
Antioxidant genes become upregulated in adult *clk-1* worms. To determine whether the upregulation of antioxidant genes could explain the resistance of *clk-1* worms to chronic oxidative stress during adulthood, we examined the time course of gene expression changes in *clk-1* worms. We examined worms at three time point: L2 worms (L2), day 1 adult worms (YA) and day 4 adult worms (A4) by quantitative real-time RT-PCR. The antioxidant genes *sod-3* (**A**), *prdx-2* (**B**), *ctl-1* (**C**) and *gcs-1* (**D**) were not upregulated at the L2 phase, but were increased in young adult worms. Thus, there is an increase in antioxidant gene expression at the start of adulthood that corresponds to the increased resistance to chronic oxidative stress in *clk-1* worms. Error bars indicate SEM. * p<0.05, ** p<0.01, *** p<0.001.

To further investigate the time course of gene expression changes in *clk-1* worms that contribute to stress resistance and longevity, we next used quantitative real-time RT-PCR to examine the expression of select antioxidant genes at three time points: the L2 stage of larval development, day 1 of adulthood and day 4 of adulthood. We found that for all three antioxidant genes tested (*sod-3*, *prdx-2* and *ctl-1*), mRNA levels were not increased in L2 worms but were upregulated during adulthood (Fig. 4A-C). We also found that the *skn-1* target *gcs-1* (gamma glutamylcysteine synthetase), which performs the rate limiting step in glutathione synthesis, was upregulated in adult *clk-1* worms (Fig. 4D). The upregulation of antioxidant genes during adulthood likely contributes to the increased resistance to chronic oxidative stress observed in *clk-1* worms.

### Elevated superoxide levels exhibit a compartment specific effect on *clk-1* lifespan

We have previously shown that *clk-1* lifespan is dramatically increased by the deletion of *sod-2* [7]. As SOD-2 encodes the primary mitochondrial *sod* gene, this indicates that increasing mitochondrial superoxide levels through decreased detoxification increases *clk-1* lifespan. To determine whether this increase in lifespan is specific to mitochondrial superoxide, or whether increased superoxide in other subcellular compartments would also increase *clk-1* lifespan, we used a genetic approach to specifically decrease superoxide detoxification in different subcellular compartments. As *C*. *elegans* have five *sod* genes [32], we generated *clk-1; sod* double mutants for the remaining four *sod* genes and measured lifespan. *sod-1*, *sod-2* and *sod-4* encode the primary cytoplasmic, mitochondrial and extracellular SODs, respectively, while *sod-3* and *sod-5* encode inducible mitochondrial and cytoplasmic SODs, which are normally expressed at low levels [33–38].

**Figure 5:**
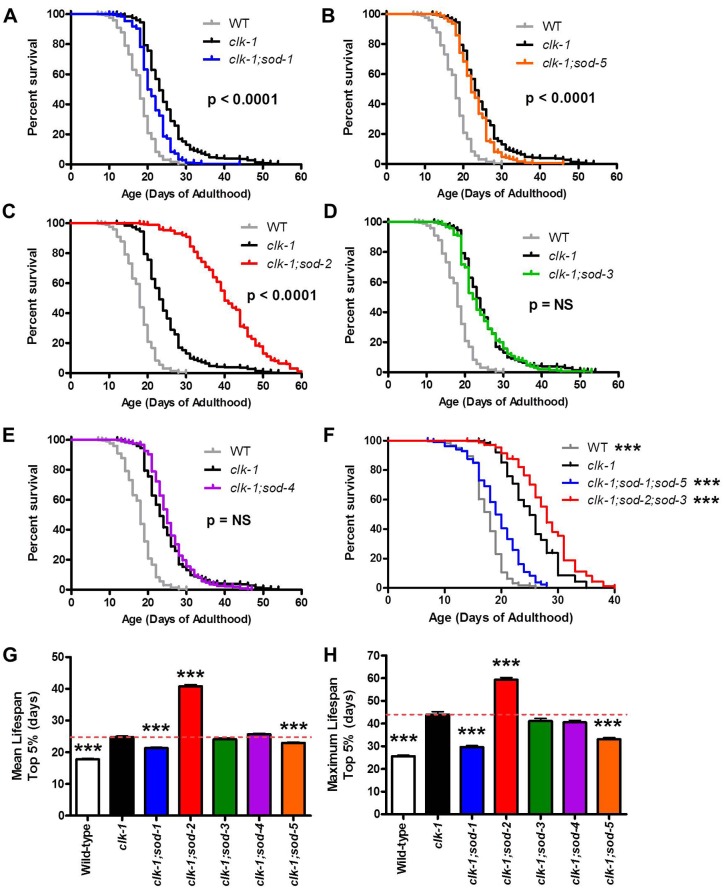
Increased superoxide has a compartment specific effect on *clk-1* lifespan. Genetic deletion of individual *sod* genes allows for a compartment specific increase in the levels of superoxide. **A,B**. Deletion of either of the cytoplasmic *sod* genes (*sod-1*, *sod-5*) decreases *clk-1* lifespan. **C**. In contrast, deletion of the primary mitochondrial *sod* gene (*sod-2*) results in a marked increase in longevity. **D,E**. Loss of the inducible mitochondrial sod gene (*sod-3*) or the extracellular *sod* gene (*sod-4*) has no effect on *clk-1* lifespan. **F**. While the loss of both cytoplasmic *sod* genes decreases *clk-1* lifespan, *clk-1;sod-2;sod-3* mutants, which have no mitochondrial matrix SOD, still live longer than *clk-1* worms. **G,H**. Mean and maximum lifespan for *clk-1* double mutants lacking individual *sod* genes. The p-values shown indicate differences from *clk-1* worms. All *clk-1* double mutants had lifespans and maximum lifespans that were significantly different from wild-type. The fact that deletion of *sod-1* or *sod-5* decreases *clk-1* lifespan, while deletion of *sod-2* increases *clk-1* lifespan demonstrates that increasing mitochondrial and cytoplasmic superoxide has opposing effects on lifespan. Error bars indicate SEM. *** p < 0.001. NS = not significant.

In contrast to the increased lifespan observed in *clk-1;sod-2* double mutants, we found that deletions in either of the cytoplasmic *sod* genes, *sod-1* or *sod-5*, resulted in a significant decrease in lifespan (Fig. 5A,B; S4A–S4B Fig.). Although the magnitude of the decrease in lifespan between *clk-1* and *clk-1;sod-5* worms is small, the difference is highly significant (p < 0.001 for mean lifespan, maximum lifespan and Log-rank test comparing survival curves). As we have previously reported, the deletion of *sod-2* dramatically increased the lifespan of *clk-1* worms (Fig. 5C; S4C Fig.). Finally, the deletion of *sod-3* or *sod-4* had no effect on the lifespan of *clk-1* worms (Fig. 5D,E; S4D–S4E Fig.).

We also examined the lifespan of *clk-1* worms with deletions in both cytoplasmic *sod* genes (*clk-1;sod-1;sod-5* worms) and both mitochondrial matrix *sod* genes (*clk-1;sod-2;sod-3* worms). We found that *clk-1;sod-1;sod-5* worms had decreased lifespan compared to *clk-1* worms, while *clk-1;sod-2;sod-3* worms have increased lifespan compared to *clk-1* worms, though the increase was less than in *clk-1;sod-2* worms (Fig. 5F). Combined these results demonstrate a compartment specific effect of ROS on lifespan: elevated superoxide levels in the mitochondria increases *clk-1* lifespan, while elevated superoxide in the cytoplasm decreases it (Fig. 5G,H).

### Compartment specific effect of ROS on stress resistance does not account for the effect of ROS on lifespan

**Figure 6:**
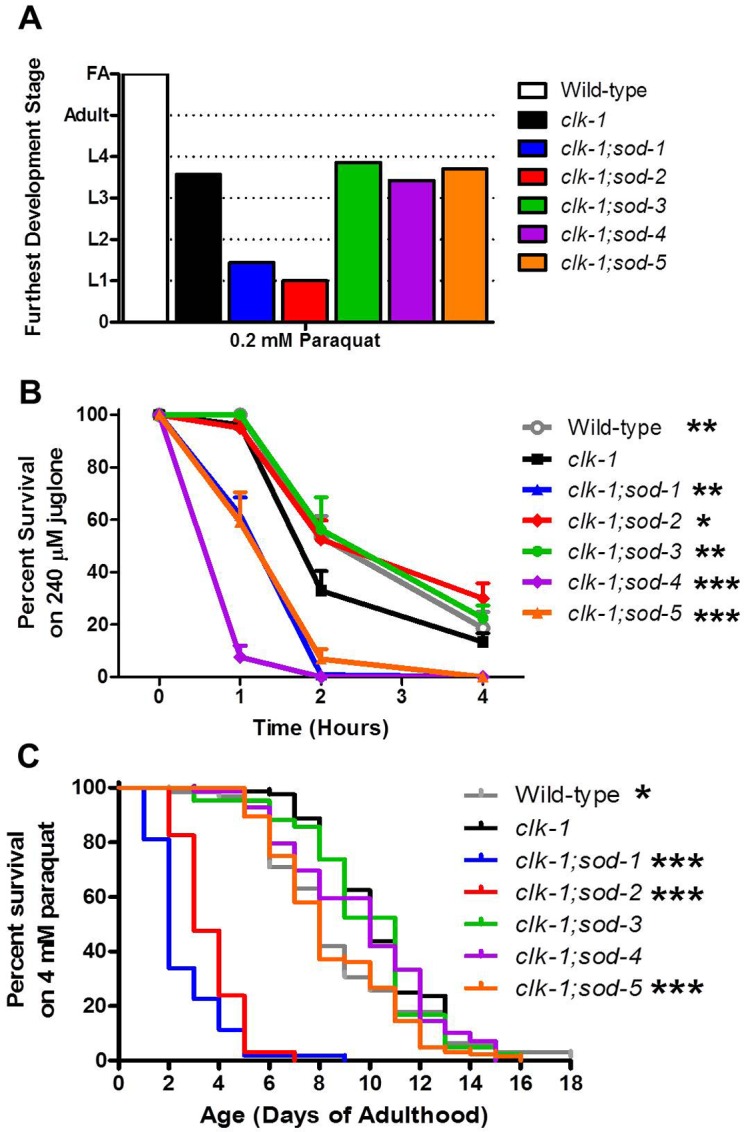
Deletion of individual *sod* genes results in a compartment specific effect on sensitivity to oxidative stress in *clk-1* worms. **A**. Deletion of *sod-1* or *sod-2* increases *clk-1* sensitivity to oxidative stress during development. **B**. Deletion of *sod-1*, *sod-4* or *sod-5* increases *clk-1* sensitivity to oxidative stress after acute exposure to juglone on day 1 of adulthood. In contrast, deletion of either mitochondrial *sod* gene, *sod-2* or *sod-3*, reverts stress sensitivity to wild-type. **C**. Deletion of *sod-1*, *sod-2* or *sod-5* increases *clk-1* sensitivity to oxidative stress during chronic exposure to 4 mM paraquat beginning on day 1 of adulthood. Note that deletion of *sod-1* and *sod-2* both increase sensitivity to paraquat in *clk-1* worms despite having opposite effects on lifespan. Significance indicates difference from *clk-1* worms. Error bars indicate SEM. * p<0.05, ** p<0.01, *** p<0.001. FA = fertile adult.

Having shown that *clk-1* lifespan is modulated by levels of ROS, we next sought to determine the extent to which the different effects on longevity could be attributed to changes in resistance to stress. If differences in stress resistance contributed to the changes in longevity observed in *clk-1;sod* double mutants, we would expect *clk-1;sod-2* mutants to have increased resistance to stress compared to *clk-1* worms and *clk-1;sod-1* and *clk-1;sod-5* worms to have decreased resistance. We measured sensitivity to oxidative stress during development, acutely during adulthood and in a chronic assay during adulthood. We found that deletion of *sod-1* or *sod-2* further increased sensitivity to oxidative stress during development in *clk-1* worms (Fig. 6A), while deletion of other *sod* genes had no effect. At this concentration of 0.2 mM paraquat, we previously showed that all of the individual *sod* deletion mutants except for *sod-2* were able to develop to adulthood [7]. Of note, *clk-1;sod-1* double mutants are more sensitive to oxidative stress during development than either single mutant.

To examine sensitivity to oxidative stress acutely on day 1 of adulthood, we measured the survival of the *clk-1;sod* double mutants on plates containing 240 μM juglone. We found that deletion of either cytoplasmic *sod* gene (*sod-1*, *sod-5*) or the extracellular *sod* gene (*sod-4*) resulted in increased sensitivity to oxidative stress in *clk-1* worms (Fig. 6B). We previously showed that deletion of *sod-1* or *sod-2* increases sensitivity to juglone on a WT background, with the greatest deficit being observed in the *sod-1* deletion mutants [7].

Finally, we examined sensitivity to oxidative stress throughout adulthood by treating worms with 4 mM paraquat beginning on day 1 of adulthood. In this assay, we found that deletion of *sod-1*, *sod-2* or *sod-5* all increased sensitivity to oxidative stress in *clk-1* worms (Fig. 6C). We have previously shown that deletion of *sod-1*, *sod-2* or *sod-3* decreases survival in this assay on a wild-type background [7].

Overall, the effect of deleting individual *sod* genes on sensitivity to oxidative stress in *clk-1* worms does not account for the observed effects on lifespan. While both *sod-1* and *sod-2* deletions markedly increase the sensitivity of *clk-1* worms to paraquat-induced oxidative stress, these *sod* genes have opposite effects on *clk-1* lifespan.

### Compartment specific effect of ROS on physiologic rates does not account for the effect of ROS on lifespan

In addition to increased lifespan, *clk-1* and other mitochondrial mutants have been shown to exhibit slow physiologic rates, which have been proposed to contribute to their longevity. To determine whether alterations in physiologic rates could account for the effect of deleting individual *sod* genes on *clk-1* lifespan, we compared the physiologic rates of *clk1;sod* double mutants to *clk-1* worms. *clk-1* worms develop significantly slower than wild-type worms. Deletion of *sod-1* or *sod-2* further lengthened the development time in *clk-1* worms while deletion of *sod-4* restored development time towards that of wild-type (S5A Fig.). Defecation cycle length is also significantly longer in *clk-1* worms compared to wild-type worms. Deletion of *sod-2* further exacerbated this phenotype while deletion of *sod-1* or *sod-4* resulted in near wild-type rates of defecation (S5B Fig.). The self-brood size of *clk-1* worms is decreased compared to wild-type worms and is further decreased by the deletion of *sod-1* or *sod-2* (S5C Fig.). In contrast, deletion of *sod-4* partially rescues the decreased brood size of *clk-1* worms (S5C Fig.). Finally, thrashing rate is decreased in *clk-1* worms compared to wild-type worms but is restored towards that of wild-type by deletion of *sod-1*, *sod-2* or *sod-4* (S5D Fig.). Thus, aside from defecation cycle length, the deletion of *sod-1* and *sod-2* has similar effects on *clk-1* physiologic rates, despite having opposite effects on lifespan. This indicates that changes in *clk-1* physiologic rates induced by deletion of individual *sod* genes cannot account for the observed differences in lifespan. Interestingly, the deletion of *sod-4*, the extracellular *sod* gene, rescues the slow development, slow defecation, decreased brood size and slow thrashing rate of *clk-1* worms, yet has no impact on *clk-1* lifespan. This provides a clear demonstration that *clk-1* physiologic rates can be experimentally dissociated from lifespan.

### Modulating ROS levels in *clk-1* worms suggests multiple mechanisms of lifespan extension

As ROS have been implicated in the longevity of *clk-1* worms [3], we next examined the effect of pharmacologic alterations of ROS levels on *clk-1* lifespan. We have previously shown that treating wild-type worms with the superoxide generator paraquat at concentrations between 0.05 and 0.5 mM increased lifespan, while paraquat concentrations of 2 mM and above resulted in decreased lifespan [4]. If elevated ROS levels in *clk-1* worms contribute to their longevity, we would predict that (1) treating *clk-1* worms with antioxidants would result in decreased lifespan and (2) *clk-1* worms would exhibit a decrease in their optimum paraquat concentration compared to wild-type worms, since ROS levels are already increased in *clk-1* worms [3,5].

**Figure 7:**
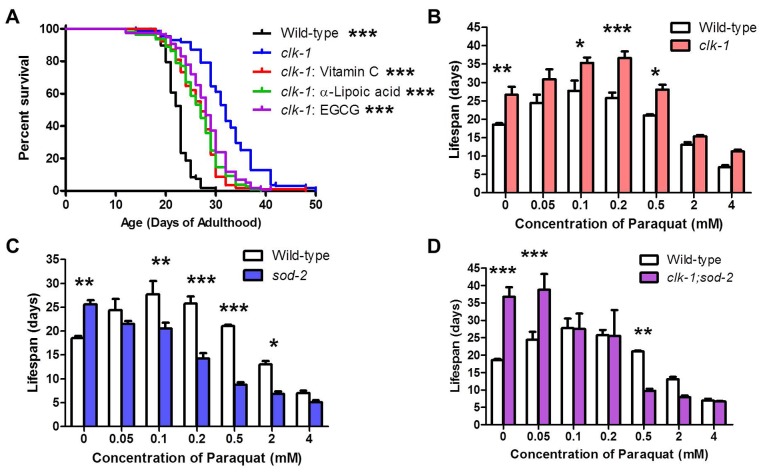
ROS-dependent and ROS-independent mechanisms contribute to longevity in *clk-1* worms. **A**. Treatment with three difference antioxidants (10 mM Vitamin C, 25 μM α-Lipoic acid or 25 μM epigallocatechin 3-gallate (EGCG) decreases *clk-1* lifespan but does not revert it to wild-type. **B**. Treatment with paraquat increases *clk-1* lifespan. The optimum paraquat concentration for *clk-1* longevity is not decreased compared to wild-type worms. **C,D**. In contrast, paraquat treatment only decreases the lifespan of *sod-2* worms and *clk-1;sod-2* worms, both of which exhibit a decrease in their optimum paraquat concentration for longevity. Significance indicates difference from *clk-1* worms. Error bars indicate SEM. *p<0.05, **p<0.01, ***p<0.001.

To test the effect of antioxidants on *clk-1* lifespan, we treated *clk-1* worms with three different antioxidants: 10 mM vitamin C, 25 μM α-lipoic acid and 25 μM epigallocatechin gallate (EGCG). In each case, we found that antioxidant treatment significantly reduced *clk-1* lifespan (Fig. 7A). However, antioxidant-treated *clk-1* worms were still long-lived compared to wild-type worms, suggesting the possibility that there are ROS-dependent and ROS-independent mechanisms involved in *clk-1* longevity. Treatment of *clk-1* worms with 10 mM vitamin C did not affect development time, brood size or thrashing rate, thereby providing further confirmation that *clk-1* longevity can be experimentally dissociated from their slow physiologic rates (S6 Fig.).

To determine the optimum level of ROS for *clk-1* lifespan, we treated *clk-1* and wild-type worms with a concentration dilution series of paraquat ranging from 0.05 mM to 4 mM. As we have previously shown, wild-type worms exhibit a maximum lifespan at 0.1 mM paraquat (Fig. 7B) [4]. Surprisingly, we found that *clk-1* worms do not exhibit a decrease in their optimum paraquat concentration with respect to lifespan. In fact, we observed the opposite. Despite having elevated levels of ROS compared to wild-type worms, we found that *clk-1* worms exhibit an increase in their optimum paraquat concentration with respect to lifespan of 0.2 mM (Fig. 7B). Comparing the pattern observed for wild-type and *clk-1* worms reveals an upward shift in the pattern for *clk-1* worms compared to wild-type.

For comparison, we examined the lifespan of *sod-2* worms exposed to the same concentration dilution series of paraquat. *sod-2* worms have increased lifespan that is due to elevated levels of mitochondrial superoxide [7]. In contrast to *clk-1* worms, treatment with paraquat does not increase the lifespan of *sod-2* worms at any concentration (Fig. 7C). As would be predicted for worms with elevated ROS, the optimum paraquat concentration with respect to lifespan is decreased compared to wild-type. It is also noteworthy that the peak lifespans for *sod-2* worms and wild-type worms treated with paraquat is similar but occur at different concentrations of paraquat, 0 mM and 0.1 mM, respectively. The fact that *clk-1* worms exhibit a distinct pattern from what is observed in *sod-2* mutants suggests that *clk-1* longevity is at least partially mediated by a ROS-independent mechanism (S7 Fig.).

As deletion of *sod-2* markedly increases the lifespan of *clk-1* worms, we next sought to determine the optimum paraquat concentration for *clk-1;sod-2* worm lifespan. As with *sod-2* worms, we found that there was no concentration of paraquat that could increase *clk-1;sod-2* lifespan (Fig. 7D). Interestingly, at very low concentrations of paraquat (0–0.05 mM paraquat), *clk-1;sod-2* worms live longer than wild-type worms, at low concentrations of paraquat they are equally long-lived while at high concentrations of paraquat (0.5 mM paraquat) wild-type worms have increased survival compared to *clk-1;sod-2* worms.

### Increasing mitochondrial ROS levels can still extend lifespan in worms with a detrimental level of cytoplasmic ROS

**Figure 8:**
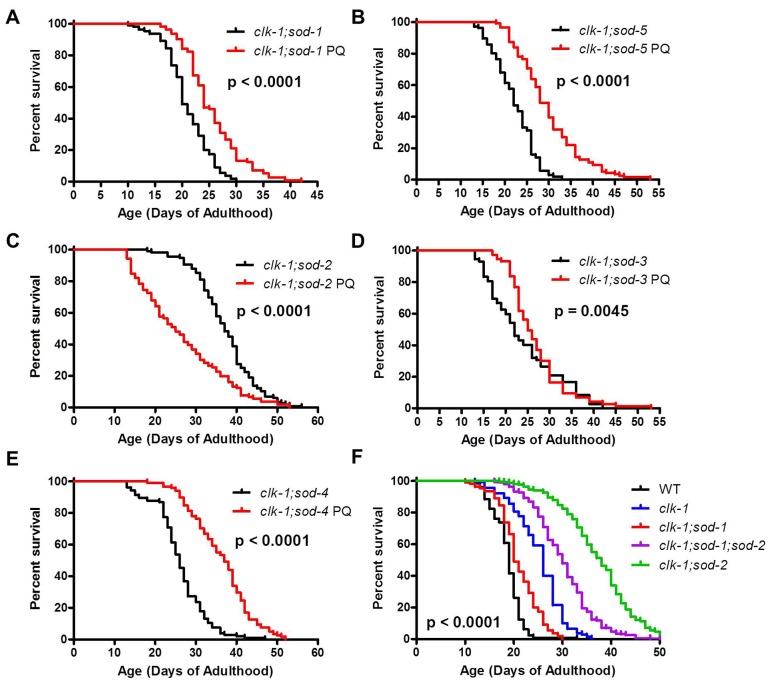
Mitochondrial superoxide increases the lifespan of worms with a detrimental level of cytoplasmic superoxide. To further test the extent to which superoxide has different effects on lifespan depending on which subcellular compartment it is in, mitochondrial superoxide levels were increased through treatment with 0.1 mM paraquat. **A,B**. Despite the fact that *clk-1;sod-1* and *clk-1;sod-5* worms have decreased lifespan compared to *clk-1* worms resulting from elevated cytoplasmic superoxde levels, both double mutants showed increased lifespan when treated with paraquat. **C**. This is in contrast to *clk-1;sod-2* worms that exhibit a decrease in lifespan when mitochondrial superoxide levels are further increased. **D,E**. *clk-1;sod-3* worms exhibited a small but significant increase in lifespan when treated with paraquat, while *clk-1;sod-4* worms exhibit a robust increase. **F**. *clk-1;sod-1;sod-2* worms show increased lifespan compared to *clk-1;sod-1* worms, but decreased lifespan compared to *clk-1;sod-2* worms. This indicates that increasing mitochondrial superoxide through the deletion of *sod-2* can increase the lifespan of *clk-1;sod-1* worms, while increasing cytoplasmic superoxide through the deletion of *sod-1* can decrease the lifespan of *clk-1;sod-2* worms. This indicates that mitochondrial and cytoplasmic superoxide have opposing effects on lifespan.

If mitochondrial and cytoplasmic superoxide have different effects on lifespan then increasing mitochondrial superoxide should still be able to increase the lifespan of *clk-1;sod-1* and *clk-1;sod-5* worms, which have decreased lifespan compared to *clk-1* worms resulting from elevated levels of cytoplasmic superoxide. To test this, we examined whether treatment with paraquat could increase the lifespan of the *clk-1;sod* double mutants. We found that, in contrast to *clk-1;sod-2* double mutants, increasing ROS levels through treatment with paraquat could increase the lifespan of all of the other *clk-1;sod* double mutants, including *clk-1;sod-1* double mutants (Fig. 8A-E, S8 Fig.). This clearly indicates that the decreased lifespan in *clk-1;sod-1* worms does not result from too much ROS. Instead, it suggests that it is high levels of ROS specifically in the cytoplasm that act to decrease *clk-1* lifespan. This result also demonstrates that increasing mitochondrial ROS can increase lifespan despite detrimental levels of cytoplasmic ROS. Conversely, the fact that *clk-1;sod-1* worms treated with paraquat have a shorter lifespan than *clk-1* worms treated with paraquat indicates that increasing cytoplasmic ROS through deletion of *sod-1* shortens the lifespan of worms that have extended longevity resulting from increased mitochondrial ROS.

To provide further support for the compartment specific effects of ROS on lifespan, we generated *clk-1;sod-1;sod-2* triple mutants, which are predicted to have increased cytoplasmic ROS compared to *clk-1;sod-2* worms and increased mitochondrial ROS compared to *clk-1;sod-1* worms. We found that *clk-1;sod-1;sod-2* worms have increased lifespan compared to *clk-1;sod-1* worms but decreased lifespan compared to *clk-1;sod-2* worms (Fig. 8F). Thus, increasing cytoplasmic ROS through the deletion of *sod-1* decreases *clk-1;sod-2* lifespan, while increasing mitochondrial ROS through the deletion of *sod-2* increases *clk-1;sod-1* lifespan. This indicates that mitochondrial and cytoplasmic ROS have opposing effects on lifespan.

## Discussion

While ROS have been proposed to be one of the main causes of aging, accumulating evidence suggests that ROS can also be beneficial. Deletion of the mitochondrial *sod* gene, *sod-2*, results in increased lifespan despite increased levels of oxidative damage[7]. In addition, treating wild-type worms with low concentrations of paraquat (paraquat), a compound that increases levels of superoxide, also results in increased lifespan[4,5]. There are now multiple examples of interventions that cause elevated ROS that also result in an increase in lifespan that is reduced by antioxidants, including treatment of worms with 2-deoxy-D-glucose[39], lonidamine[40], arsenic[41], complex I inhibitors[42], or D-glucosamine[43]. Similarly, mutations in the insulin-IGF1 receptor gene *daf-2*[8]; overexpression of sirtuin (*sir-2*.*1*)[44]; and mutations in genes that affect electron transport chain function (*clk-1*, *isp-1*, and *nuo-6*)[9,45,46] have been shown to increase both ROS levels and lifespan. Importantly, the ability of ROS to increase lifespan is conserved across species. Yeast treated with menadione to elevate mitochondrial ROS have increased lifespan[47]. As in worms, mice with a mutation in the *clk-1* ortholog *Mclk1*[48] and mice treated with D-glucosamine[43] exhibit elevated ROS levels and increased lifespan. In order to elucidate the mechanism by which ROS act to increase lifespan, it will be important to define the levels and localization of ROS that are required to promote longevity.

### Compartment specific effect of ROS on lifespan

To gain insight into the sub-cellular localization requirements for ROS to increase lifespan, we used a genetic approach to specifically increase ROS in different sub-cellular compartments in *clk-1* worms, which have increased sensitivity to ROS. As it is currently not possible to detect levels of superoxide in a specific subcellular compartment in a living worm, we were unable to show directly that deletion of the *sod* genes results in increased superoxide levels in the compartment in which the gene was expressed. However, our previous work showing increased sensitivity to oxidative stress and increased oxidative damage in the *sod* deletion mutants suggests that superoxide levels are increased. Since superoxide is not able to cross biological membranes[49], any increase in superoxide levels should be limited to the subcellular compartment in which the *sod* gene was normally expressed.

While deletion of the primary mitochondrial *sod* gene (*sod-2*) results in a dramatic increase in *clk-1* lifespan, loss of either cytoplasmic *sod* gene (*sod-1* or *sod-5*) significantly decreased *clk-1* lifespan. This is particularly surprising for *sod-5*, which is normally expressed at very low levels[50], and is only mildly upregulated in *clk-1* worms. Deletion of *sod-1* has previously been shown to have either no impact or a small negative impact on lifespan on a wild-type background, while *sod-5* has not been shown to affect longevity [7,50,51]. The deletion of *sod-3*, the inducible mitochondrial *sod* gene, or *sod-4*, the extracellular *sod* gene, had no impact on lifespan in *clk-1* or wild-type worms. While *sod-3* is expressed in the same subcellular compartment as *sod-2*, it is likely that deletion of *sod-3* does not increase mitochondrial superoxide levels enough to increase *clk-1* lifespan as *sod-3* normally accounts for only 1% of the total *sod* mRNA in a cell [50]. Overall these results indicate that elevated superoxide levels act specifically in the mitochondria, and not other sub-cellular compartments, to increase lifespan. Interestingly, mice that are heterozygous for the targeted inactivation of the mouse ortholog of *clk-1* (*Mclk1*), which also exhibit extended longevity [52], have increased oxidative damage in the mitochondria and decreased damage in the cytoplasm in young mice [53]. Based on our present results, both the increased mitochondrial ROS and decreased cytoplasmic ROS could contribute to the extended lifespan of *Mclk1+/-* mice.

### Multiple mechanisms of lifespan extension in *clk-1* worms

To specifically test the role of elevated ROS in the longevity of *clk-1* worms, we examined the effect of pharmacologically increasing and decreasing ROS levels on *clk-1* lifespan. In contrast to the predictions of the free radical theory of aging, we found that decreasing ROS decreased *clk-1* lifespan, while increasing ROS increased *clk-1* lifespan. Treating *clk-1* worms with three different antioxidants (vitamin C, α-lipoic acid and epigallocatechin gallate) all resulted in a similar partial reduction of *clk-1* lifespan. This suggests that the increased ROS levels in *clk-1* worms are required for their full longevity. The fact that the anti-oxidant treated lifespan was still longer than wild-type suggests the possibility that there are also ROS-independent mechanisms contributing to *clk-1* longevity. A previous study examined the effect of a different antioxidant, N-acetyl cysteine (NAC), on *clk-1* lifespan and observed no decrease [3]. While it is unclear why treatment with NAC did not decrease *clk-1* lifespan, it may have been due to differences in the mechanism of action between the different antioxidants, due to the fact that NAC affects bacterial growth, due to differences in experimental conditions or due to non-antioxidant effects of NAC on lifespan.

Since *clk-1* worms have elevated levels of ROS at baseline, we predicted that their optimum superoxide concentration would be decreased compared to wild-type worms. However, we observed that the maximum lifespan for *clk-1* worms occurred at a concentration of paraquat that was higher than the optimum concentration for wild-type worms with respect to longevity. This was in stark contrast to what we observed in *sod-2* deletion mutants where even small increases in superoxide levels resulted in a decrease in lifespan. The differing response of *clk-1* and *sod-2* worms to paraquat treatment suggests that different mechanisms are responsible for their longevity. Combined with our observation that antioxidant treatment decreases *clk-1* lifespan, this suggests that *clk-1* has both ROS-dependent and ROS-independent contributions to their longevity.

### Compartment specific effect of ROS on stress resistance and physiologic rates

In addition to having a compartment specific effect on lifespan, we also found that ROS have a compartment specific effect on both sensitivity to oxidative stress and physiologic rates (summarized in S1 Table). However, the effect of *sod* gene deletions on physiologic rates or sensitivity to oxidative stress was in no case predictive of the effect on lifespan. Relative to *clk-1* worms, both *clk-1;sod-1* and *clk-1;sod-2* worms have slow development, decreased brood size, arrestment of larval development under conditions of oxidative stress and increased sensitivity to chronic oxidative stress during adulthood. Thus, deletion of either *sod-1* or *sod-2* in *clk-1* worms results in similar changes in physiologic rates and stress sensitivity despite the fact that they have opposite effects on lifespan. This indicates that stress resistance and physiologic rates can be experimentally dissociated from longevity.

In examining the effect of increased ROS on the already slow physiologic rates of *clk-1* worms, we found that the deletion of *sod-4* partially restores all of the *clk-1* phenotypes towards wild-type except for lifespan. This finding is particularly interesting given the fact that deletion of *sod-4* does not alter physiologic rates on a wild-type background [4]. The fact that *sod-4* deletion rescues development rate, defecation rate, brood size and thrashing rate but does not affect *clk-1* lifespan indicates that the slowing of other physiologic rates is not necessary for the extended lifespan of *clk-1* worms. Conversely, we observed that treating *clk-1* worms with vitamin C had no effect on development time, brood size or thrashing rate, but significantly decreased *clk-1* lifespan towards wild-type. Combined, these findings indicate that independent mechanisms are responsible for the increased longevity and slow physiologic rates in *clk-1* worms.

### Upregulation of antioxidant genes during adulthood causes increased resistance to chronic oxidative stress

In examining the sensitivity of *clk-1* worms to oxidative stress, we found that sensitivity to oxidative stress was dependent on the age of the worms and whether it was an acute or chronic exposure to stress. During larval development and with acute exposure to oxidative stress during adulthood, *clk-1* worms have increased sensitivity to oxidative stress. However, in a chronic assay throughout adulthood, *clk-1* worms exhibit increased resistance to oxidative stress. This is consistent with observations of decreased oxidative damage in *clk-1* worms [14,19,21,22,54]. We found that the change in resistance to oxidative stress corresponds to the upregulation of antioxidant genes. During larval development, we found that the expression of representative antioxidant genes (*sod-3*, *prdx-2*, *ctl-1*) were equally expressed in *clk-1* and wild-type worms. In contrast, adult *clk-1* worms have increased expression of *sod*, *prdx*, *ctl* and some *trx* genes, demonstrating a broad upregulation of antioxidant defense. Thus, it is plausible that altered electron transport chain function in *clk-1* worms leads to elevated levels of ROS, which result in increased sensitivity to oxidative stress early in life but also trigger the upregulation of antioxidant defense genes that compensate for the increased level of ROS production leading to an increased resistance to chronic exposure to oxidative stress during adulthood. We also show that *clk-1* worms have increased activity of a *gst-4* reporter construct and increased *gcs-1* mRNA levels. As both *gst-4* and *gcs-1* are targets of *skn-1*, this indicates that phase II detoxification pathways are also upregulated in *clk-1* worms and likely also contribute to their resistance to oxidative stress during adulthood. Our results are in agreement with a previous microarray experiment showing increased expression of antioxidant genes in *clk-1* worms including *sod-3*, *gst-4* and *gst-8*[27].

### Conclusions

Overall, we show that mitochondrial superoxide increases *clk-1* lifespan, while cytoplasmic superoxide decreases it. Combined with our previous results showing that mild elevation of mitochondrial superoxide levels can increase lifespan, while high levels of superoxide are toxic [4], this clearly indicates that the effect of superoxide on lifespan is dependent on where and how much superoxide is present. Our data demonstrates that *clk-1* worms have increased expression of antioxidant defenses during adulthood that results in resistance to chronic exposure to oxidative stress, and suggest that there are ROS-dependent and ROS-independent mechanisms contributing to *clk-1* longevity (S9 Fig.).

## Materials and Methods

### Strains

The following strains were used in these experiments: N2 (wild-type), *sod-1(tm776)*, *sod-1(tm783)*, *sod-2(gk257)*, *sod-2(ok1030)*, *sod-3(tm760)*, *sod-4(gk101)*, *sod-5(tm1146)*, *sod-5(tm1246)*, *clk-1(qm30)*, *isp-1(qm150)*. Strains obtained from external sources were outcrossed with our N2 worms for a minimum of 6 generations. For these experiments the following double and triple mutant strains were generated: *clk-1(qm30);sod-1(tm776)*, *clk-1(qm30);sod-1(tm783)*, *clk-1(qm30);sod-2(ok1030)*, *clk-1(qm30);sod-3(tm760)*, *clk-1(qm30);sod-4(gk101)*, *clk-1(qm30);sod-5(tm1146)*, *clk-1(qm30);sod-5(tm1246)*, *clk-1(qm30);sod-1(tm783);sod-2(ok1030)*, *clk-1(qm30);sod-2(ok1030);sod-3(tm760)*. We did not observe any phenotypic differences between *clk-1(qm30);sod-1(tm776)* and *clk-1(qm30);sod-1(tm783)* or *clk-1(qm30);sod-5(tm1146)* and *clk-1(qm30);sod-5(tm1246)* worms. All of the *sod* deletions and the *clk-1* deletion were confirmed by PCR. All strains were maintained at 20°C.

### Lifespan analysis

Lifespan studies were completed at 20°C with a minimum of 3 independent trials and an initial number of 80 worms per strain per trial on plates containing 100 μM FUdR (Sigma). Survival plots shown represent pooled data from multiple trials. For lifespan assays involving 10 mM vitamin C, 25 μM α-lipoic acid, 25 μM epigallocatechin gallate or 0.05–4 mM paraquat, concentrated solutions of the chemical to be added were prepared fresh on the day that the plates were made. The chemical was added to the plates just prior to pouring. Plates were made fresh weekly. In assessing the effect of antioxidants on lifespan, we planned to include 10 mM N-acetyl cysteine (NAC). However, we observed abnormal bacterial growth on these plates, which had the potential to confound the lifespan results. Accordingly, lifespan was tested only on the three antioxidants listed above.

### Paraquat and juglone sensitivity assays

Juglone sensitivity assays were completed in triplicate with 30–40 worms per strain per trial at 20°C on plates containing 180–300 μM juglone (Sigma). For this assay, plates were made fresh on the day of the assay as the toxicity of juglone decreases rapidly over time (S1B Fig.). Worms that developed on NGM plates were transferred to juglone plates at either the L2 or young adult stage and survival was monitored for 6 hours. To assess the ability of worms to develop under oxidative stress, a minimum of 40 eggs were placed on plates containing 0.1–0.25 mM paraquat and seeded with OP50 bacteria. The furthest developmental stage reached was recorded. As a chronic assay of oxidative stress, 20 worms per strain per trial were grown to adulthood on NGM plates and then transferred to plates containing 4 mM paraquat at the young adult stage (day 1 of adulthood). The plates contained 100 μM FUdR to prevent paraquat-induced internal hatching of progeny. Survival was monitored daily. To assess sensitivity to acute oxidative stress using paraquat, worms that were grown on NGM plates were transferred to plates containing 200 mM paraquat at either the L2 or young adult stage. Survival was monitored hourly for a period of 15 hours. For each assay a minimum of 3 biological replicates was completed.

### Quantitative real-time RT-PCR

RNA was isolated from young adult worms using TRIZOL reagent (Invitrogen). Subsequently, 1 μg of RNA was converted to cDNA using the Quantitect Reverse Transcription kit (Qiagen). 1 μl of the resulting cDNA preparation was used for quantitative real-time PCR using the Quantitect SYBR Green PCR kit and a Biorad iCycler RT-PCR machine. Results represent the average of at least three independent biological samples.

### Measurement of fluorescent reporter activity

A Cellomics arrayscan high-content imager was used to measure reporter activity in larval worms. Worms were suspended in M9 buffer, immobilized with levamisole to a final concentration of 2 mM and transferred to a 96-well dish at a density such that worms were not touching. Brightfield and fluorescent images were captured by the imager and used to quantify whole worm fluorescence. Reporter activity in adult worms was assessed through measurement of whole worm florescence. For each of 3 biological replicates, approximately 10 worms were paralyzed with levamisole (2mM), mounted on an NGM plate and florescent images were captured using an AVT Stingray F145B camera and VimbaViewer 1.1.2 software. ImageJ was used to measure the average pixel intensity.

### Post-embryonic development

Eggs were collected and allowed to hatch over a period of 3 hours. After 3 hours, L1 worms were transferred to a new plate and monitored for development to an adult worm. Results are the average of at least three independent trials with 20 worms per trial.

### Defecation

Defecation cycle length in young adult worms was measured as the average time between consecutive pBoc contractions. Results represent a minimum of 3 trials with 10 worms per trial.

### Self-brood size

To determine the average number of progeny produced by each strain, L4 worms were placed on individual NGM plates. Worms were transferred daily until egg laying ceased and the total number of live progeny produced was counted.

### Statistical analysis

Survival plots were compared using the log-rank test. The maximum lifespan of a given strain was measured as the average of the lifespan of the longest-living 5% of worms of that strain. A one way ANOVA was used to compare mean and maximum lifespan between strains. For analyses involving multiple groups and time points, a two way ANOVA was used to assess significance followed by Bonferroni post-hoc tests for detecting specific differences between groups.

## Supporting Information

S1 Fig *clk-1* worms are sensitive to oxidative stress during development. **A**. Sensitivity to oxidative stress was assessed by transferring eggs from WT and *clk-1* worms to plates containing increasing concentrations of paraquat (PQ). While WT worms can develop to adulthood at paraquat concentrations up to 0.4 mM, *clk-1* worms arrest during larval development beginning at 0.2 mM paraquat. This indicates that *clk-1* worms are sensitive to oxidative stress during development. **B**. The toxicity of juglone plates decreases rapidly with time. *sod-1;sod-2;sod-4;sod-4;sod-5* (*sod-12345*) worms were transferred to 300 μM juglone plates at 1, 2, 3, 4, 5, 6, 7 and 8 hours after the plates were poured. The survival of the *sod-12345* worms was measured 1 hour later. Within 5 hours of the plates being poured *sod-12345* worms exhibited close to 0% survival after 1 hour. However, by 8 hours after the plates were poured, *sod-12345* worms exhibited close to 100% survival. This indicates that the toxicity of juglone plates decreases rapidly over the course of 8 hours. As a result, it would be unfeasible to use juglone for chronic oxidative stress assays.
(TIF)

S2 Fig *clk-1* worms are sensitive to acute exposure to oxidative stress throughout adulthood. *clk-1* and wild-type worms were treated with three different concentrations of juglone (180, 240 and 300 μM) on days 3, 5, 8, and 12 of adulthood. At every time point and concentration tested, *clk-1* worms exhibited decreased survival compared to wild-type worms. This indicates that *clk-1* worms have increased sensitivity to acute exposure to oxidative stress during adulthood.
(TIF)

S3 Fig The ability of worms to engage oxidative stress response is lost with increasing age. A *Pgst4*::*GFP* reporter strain was used to test the ability of worms to respond to oxidative stress with increasing age on a wild-type and *clk-1* background. WT worms that do not express the *Pgst-4*::*GFP* reporter were included as a control for autofluorescence. At day 1 and day 10 of adulthood worms were transferred to 2 mM paraquat or control plates for 24 hours and then imaged. WT worms exhibited a small degree of autofluorescence at day 10. In the wild-type background, day 1 adult worms exhibited a marked increase in *Pgst-4* reporter activity after treatment with paraquat. However, by day 10 of adulthood the worms showed no activation. In contrast, *clk-1* worms have increased *Pgst-4*::*GFP* reporter activity on day 1 of adulthood compared to control *Pgst-4*::*GFP* worms but do not show a further increase in reporter activity upon treatment with paraquat. As with *Pgst-4*::*GFP* worms, *clk-1;Pgst-4*::*GFP* worms do not show reporter activation at day 10 of adulthood after treatment with paraquat. Error bars indicate SEM. *** p<0.001.
(TIF)

S4 Fig Increased superoxide has a compartment specific effect on *clk-1* lifespan (includes individual *sod* deletion mutant lifespans). Genetic deletion of individual *sod* genes allows for a compartment specific increase in the levels of superoxide. **A,B**. Deletion of either of the cytoplasmic *sod* genes (*sod-1*, *sod-5*) *decreases clk-1* lifespan. **C**. In contrast, deletion of the primary mitochondrial *sod* gene (*sod-2*) results in a marked increase in longevity. **D,E**. Loss of the inducible mitochondrial sod gene (*sod-3*) or the extracellular *sod* gene (*sod-4*) has no effect on *clk-1* lifespan. The p-values shown indicate differences from *clk-1* worms. All *clk-1* double mutants had lifespans and maximum lifespans that were significantly different from wild-type. The fact that deletion of *sod-1* or *sod-5* decreases *clk-1* lifespan, while deletion of *sod-2* increases *clk-1* lifespan demonstrates that increasing mitochondrial and cytoplasmic superoxide has opposing effects on lifespan. Error bars indicate SEM. *** p < 0.001. NS = not significant.
(TIF)

S5 Fig Deletion of individual *sod* genes has compartment specific effect on physiological rates of *clk-1* worms. **A**. *clk-1* worms develop slower than wild-type worms. The post-embryonic development time of *clk-1* worms was further slowed by deletion of *sod-1* or *sod-2* but partially rescued by deletion of *sod-4*. **B**. The defecation cycle length of *clk-1* worms is slower than wild-type worms. This phenotype is exacerbated by deletion of *sod-2* and rescued by deletion of *sod-1* or *sod-4*. **C**. *clk-1* worms have decreased brood size compared to wild-type worms. *clk-1* brood size is further decreased by deletion of *sod-1* or *sod-2* but restored towards wild-type by deletion of *sod-4*. **D**. The thrashing rate of *clk-1* worms is slower than wild-type worms and is restored towards wild-type by deletion of *sod-1*, *sod-2* or *sod-4*. Significance indicates difference from *clk-1* worms. Error bars indicate SEM. *p<0.05, **p<0.01, ***p<0.001.
(TIF)

S6 Fig Treatment with antioxidant Vitamin C does not rescue slow physiologic rates in *clk-1* worms. *clk-1* worms were treated with 10 mM vitamin C beginning at the L4 stage. Physiologic rates were assessed in the F1 progeny. This concentration of vitamin C was sufficient to decrease *clk-1* lifespan **A**. Treatment with vitamin C did not affect the development time of *clk-1* or WT worms. **B**. Treatment with vitamin C partially rescued the slow defecation cycle length in *clk-1* worms but had no effect in WT worms. **C**. Treatment with vitamin C significantly decreased the brood size of WT worms but not *clk-1* worms. **D**. Thrashing rate was not affected by treatment with vitamin C. Error bars indicate SEM. * p<0.05, *** p<0.001.
(TIF)

S7 Fig Differential response of *clk-1* and *sod-2* worms to paraquat suggests independent mechanisms of lifespan extension. **A,B**. As in WT worms, treatment of *clk-1* worms with low concentrations of paraquat results in increased lifespan. In contrast, treating *sod-2* worms with paraquat only results in decreased lifespan. As the longevity of *sod-2* worms results from elevated mitochondrial superoxide, this suggests that *clk-1* worms also have a ROS-independent mechanism of lifespan extension. Interestingly, the maximum lifespan across the concentration series is similar for *clk-1* worms and *clk-1;sod-2* worms, as it is for WT and *sod-2* worms. In both cases, the optimum concentration of paraquat is decreased by the *sod-2* mutation. The presence of the *clk-1* mutation increases lifespan above the maximum lifespans achieved by *sod-2* deletion or treatment with paraquat alone. **C**. As with paraquat, increasing ROS through treatment with 2-deoxyglucose (DOG) increases the lifespan of *clk-1* and WT worms. Error bars indicate SEM. *** p<0.001.
(TIF)

S8 Fig Paraquat increases the lifespan of all of the *clk-1;sod* double mutants except for *clk-1;sod-2*. Treatment with low concentrations of paraquat (0.1 mM) is able to extend the lifespan of WT, *clk-1*, *clk-1;sod-1*, *clk-1;sod-3*, *clk-1;sod-4* and *clk-1;sod-5* double mutants. In contrast, the same treatment resulted in a significant reduction in *clk-1;sod-2* lifespan. Error bars indicate SEM. *p<0.05, **p<0.01, ***p<0.0001.
(TIF)

S9 Fig Model for increased longevity in *clk-1* worms. The *clk-1* mutation leads to decreased levels of oxidative phosphorylation and increased production of ROS. Elevated ROS cause increased sensitivity to oxidative stress and leads to increased expression of antioxidant genes. The increase in antioxidant gene expression results in increased resistance to chronic oxidative stress and decreased oxidative damage. Elevated ROS in *clk-1* worms also activate stress-responsive pathways (e.g. hypoxic response) that contribute to *clk-1* longevity. The slow physiologic rates in *clk-1* worms can be experimentally dissociated from their longevity and likely result from decreased energy utilization, as *clk-1* worms have normal levels of ATP despite decreased energy production. Altered metabolism also contributes to *clk-1* longevity. Grey arrows indicate hypothetical connections. Red text indicates genes that are required for the associated arrow.
(TIF)

S1 Table The effect of *sod* gene deletion on lifespan, resistance to oxidative stress and physiologic rates. Black text indicates the effect of *sod* gene deletion on wild-type worms. Red text indicates the effect of *sod* gene deletion in *clk-1* worms. Red highlighting indicates *sod* gene deletion resulting in an exacerbation of the *clk-1* phenotype. Green highlighting indicates *sod* gene deletion resulting in an amelioration of the *clk-1* phenotype. Up arrows indicate increase, down arrows indicate decrease, = indicate no change. Date compiled from Figs. 5, 6 and S6.
(TIF)
