# Supplementary figures and images for: Mitochondrial and Cytoplasmic ROS Have Opposing Effects on Lifespan
Source: PLoS Genet. 2015 Feb 11;11(2):e1004972. doi: 10.1371/journal.pgen.1004972 (PMC4335496; doi:10.1371/journal.pgen.1004972)

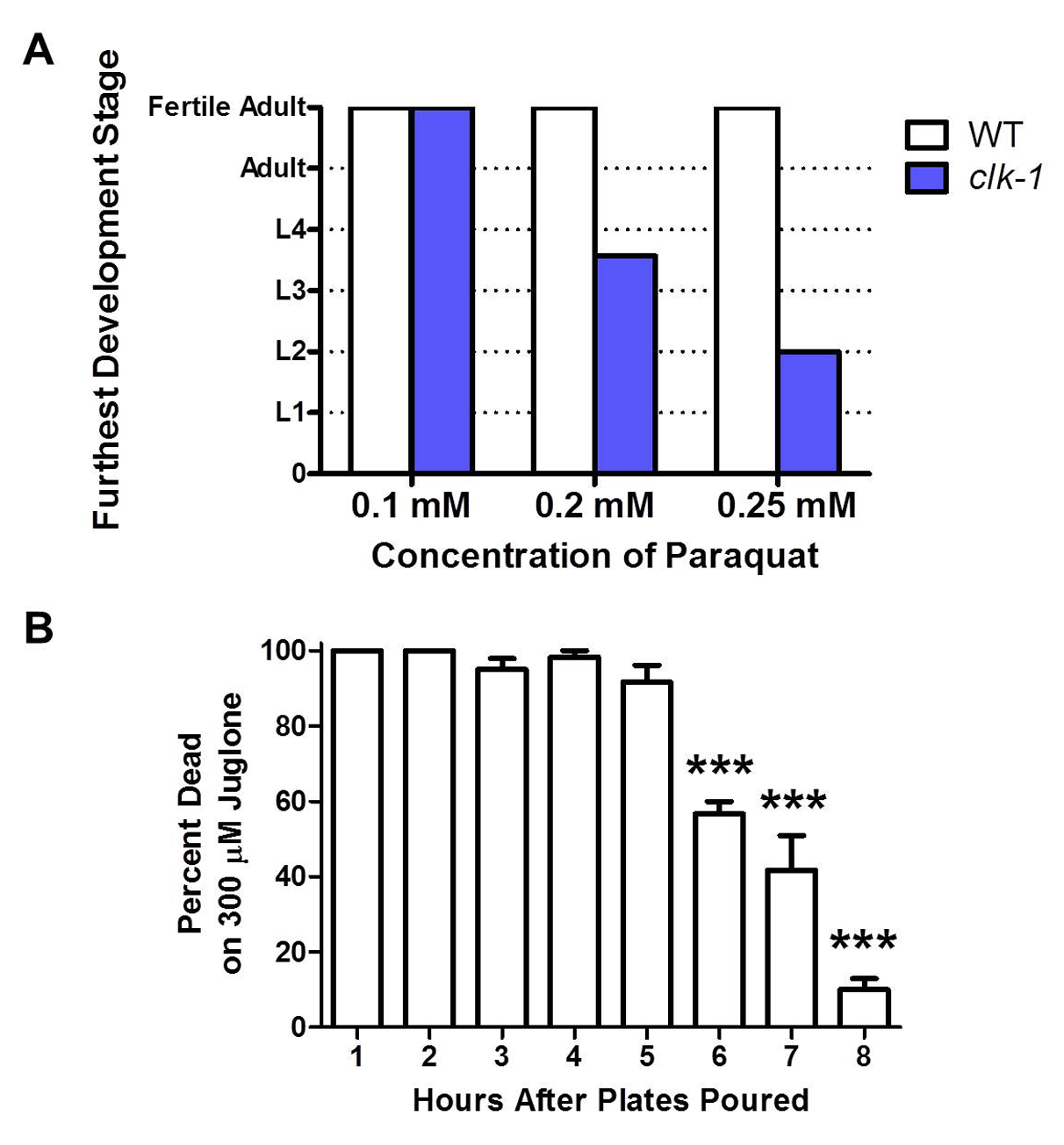

Supplement: S1 Fig — A. Sensitivity to oxidative stress was assessed by transferring eggs from WT and clk-1 worms to plates containing increasing concentrations of paraquat (PQ). While WT worms can develop to adulthood at paraquat concentrations up to 0.4 mM, clk-1 worms arrest during larval development beginning at 0.2 mM paraquat. This indicates that clk-1 worms are sensitive to oxidative stress during development. B. The toxicity of juglone plates decreases rapidly with time. sod-1;sod-2;sod-4;sod-4;sod-5 (sod-12345) worms were transferred to 300 μM juglone plates at 1, 2, 3, 4, 5, 6, 7 and 8 hours after the plates were poured. The survival of the sod-12345 worms was measured 1 hour later. Within 5 hours of the plates being poured sod-12345 worms exhibited close to 0% survival after 1 hour. However, by 8 hours after the plates were poured, sod-12345 worms exhibited close to 100% survival. This indicates that the toxicity of juglone plates decreases rapidly over the course of 8 hours. As a result, it would be unfeasible to use juglone for chronic oxidative stress assays. (TIF) [file pgen.1004972.s001.tif]

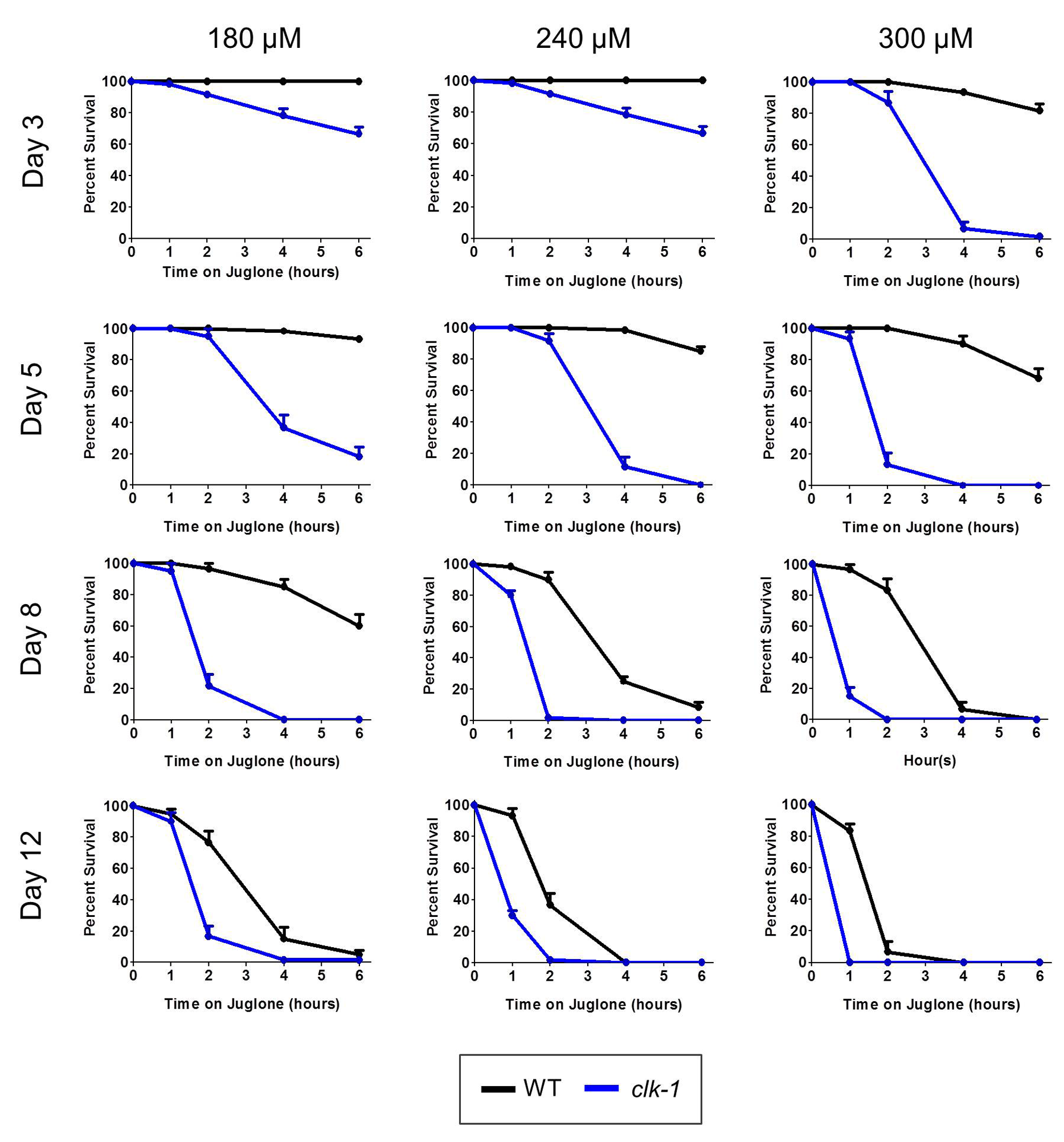

Supplement: S2 Fig — clk-1 and wild-type worms were treated with three different concentrations of juglone (180, 240 and 300 μM) on days 3, 5, 8, and 12 of adulthood. At every time point and concentration tested, clk-1 worms exhibited decreased survival compared to wild-type worms. This indicates that clk-1 worms have increased sensitivity to acute exposure to oxidative stress during adulthood. (TIF) [file pgen.1004972.s002.tif]

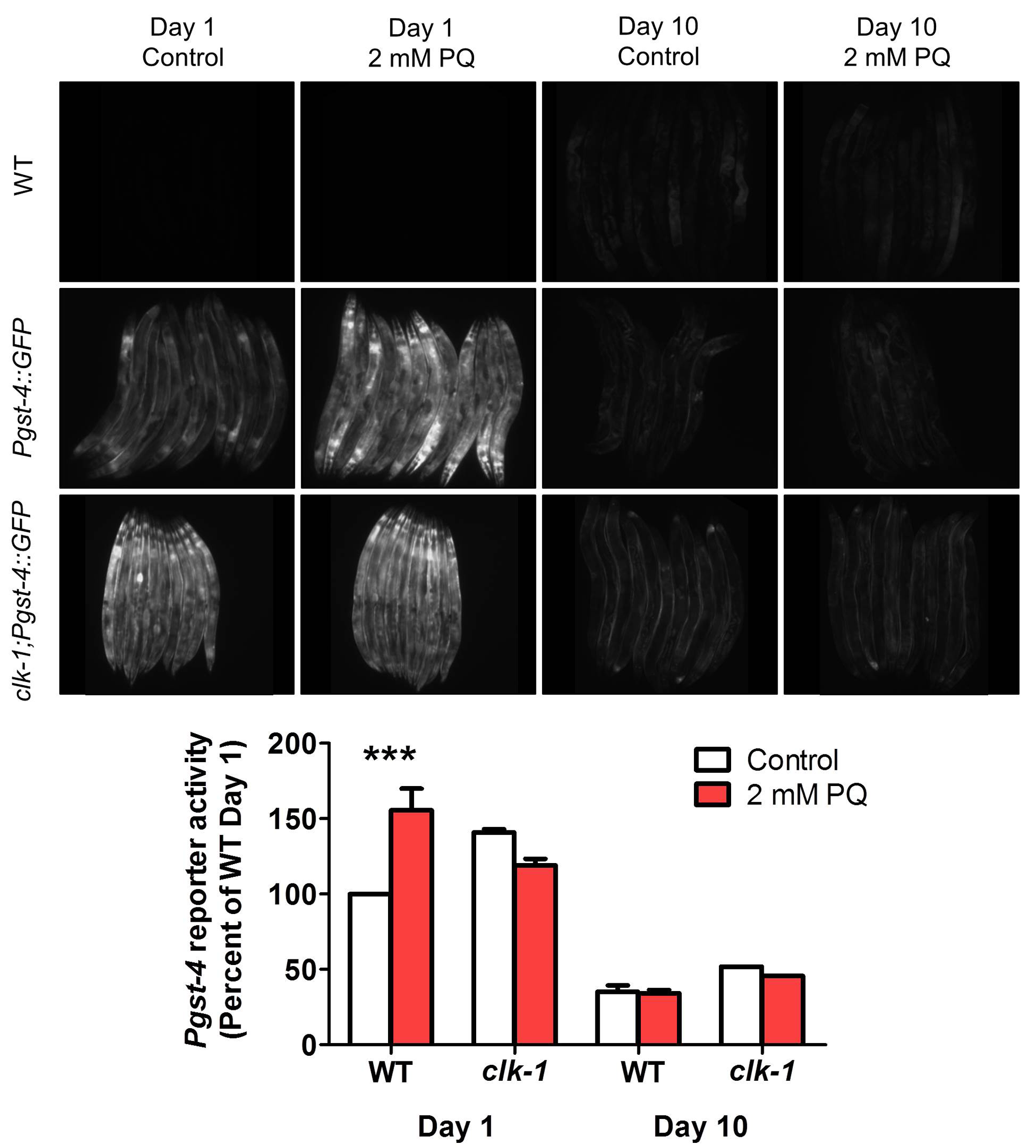

Supplement: S3 Fig — A Pgst4::GFP reporter strain was used to test the ability of worms to respond to oxidative stress with increasing age on a wild-type and clk-1 background. WT worms that do not express the Pgst-4::GFP reporter were included as a control for autofluorescence. At day 1 and day 10 of adulthood worms were transferred to 2 mM paraquat or control plates for 24 hours and then imaged. WT worms exhibited a small degree of autofluorescence at day 10. In the wild-type background, day 1 adult worms exhibited a marked increase in Pgst-4 reporter activity after treatment with paraquat. However, by day 10 of adulthood the worms showed no activation. In contrast, clk-1 worms have increased Pgst-4::GFP reporter activity on day 1 of adulthood compared to control Pgst-4::GFP worms but do not show a further increase in reporter activity upon treatment with paraquat. As with Pgst-4::GFP worms, clk-1;Pgst-4::GFP worms do not show reporter activation at day 10 of adulthood after treatment with paraquat. Error bars indicate SEM. *** p<0.001. (TIF) [file pgen.1004972.s003.tif]

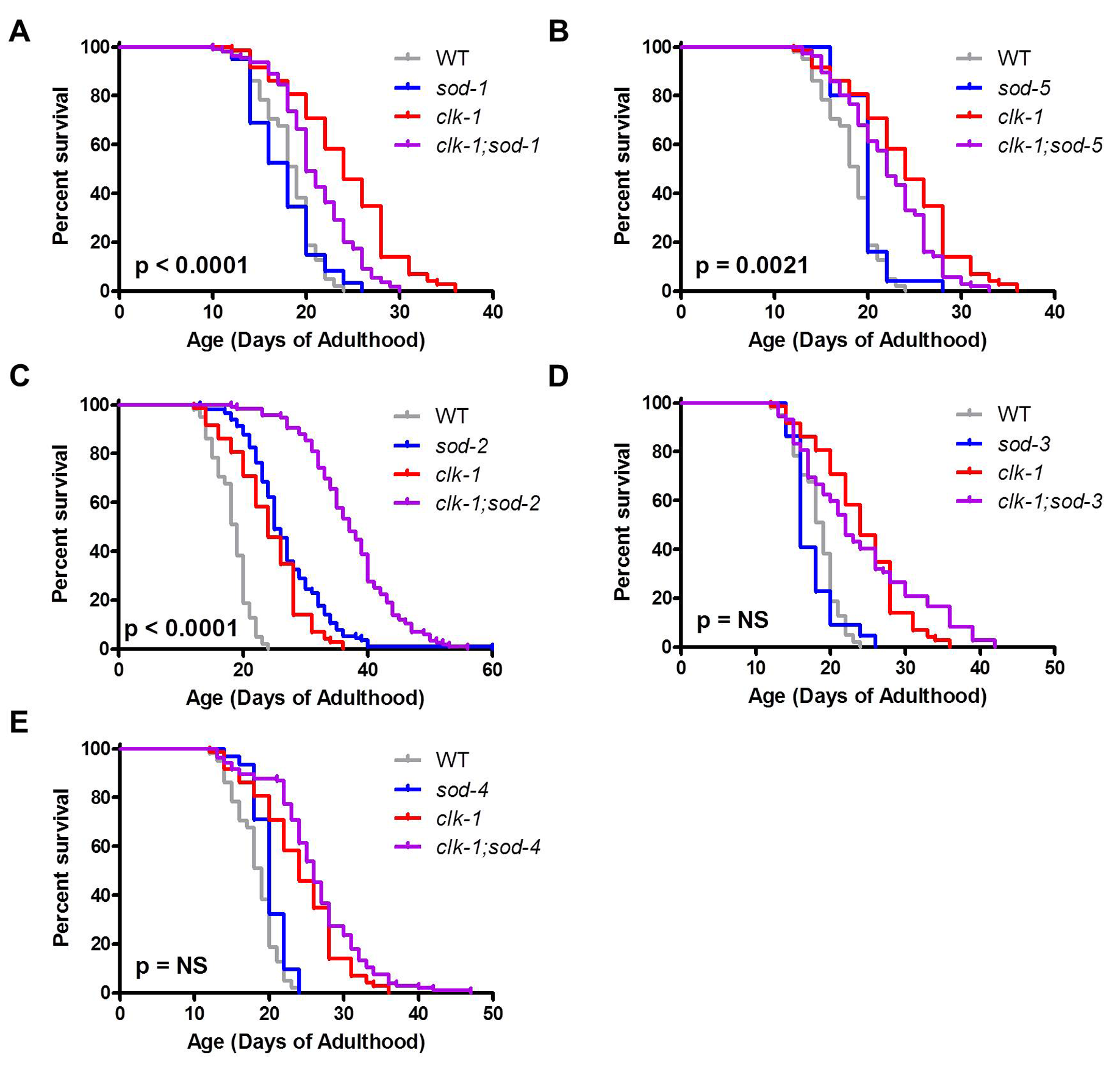

Supplement: S4 Fig — Genetic deletion of individual sod genes allows for a compartment specific increase in the levels of superoxide. A,B. Deletion of either of the cytoplasmic sod genes (sod-1, sod-5) decreases clk-1 lifespan. C. In contrast, deletion of the primary mitochondrial sod gene (sod-2) results in a marked increase in longevity. D,E. Loss of the inducible mitochondrial sod gene (sod-3) or the extracellular sod gene (sod-4) has no effect on clk-1 lifespan. The p-values shown indicate differences from clk-1 worms. All clk-1 double mutants had lifespans and maximum lifespans that were significantly different from wild-type. The fact that deletion of sod-1 or sod-5 decreases clk-1 lifespan, while deletion of sod-2 increases clk-1 lifespan demonstrates that increasing mitochondrial and cytoplasmic superoxide has opposing effects on lifespan. Error bars indicate SEM. *** p < 0.001. NS = not significant. (TIF) [file pgen.1004972.s004.tif]

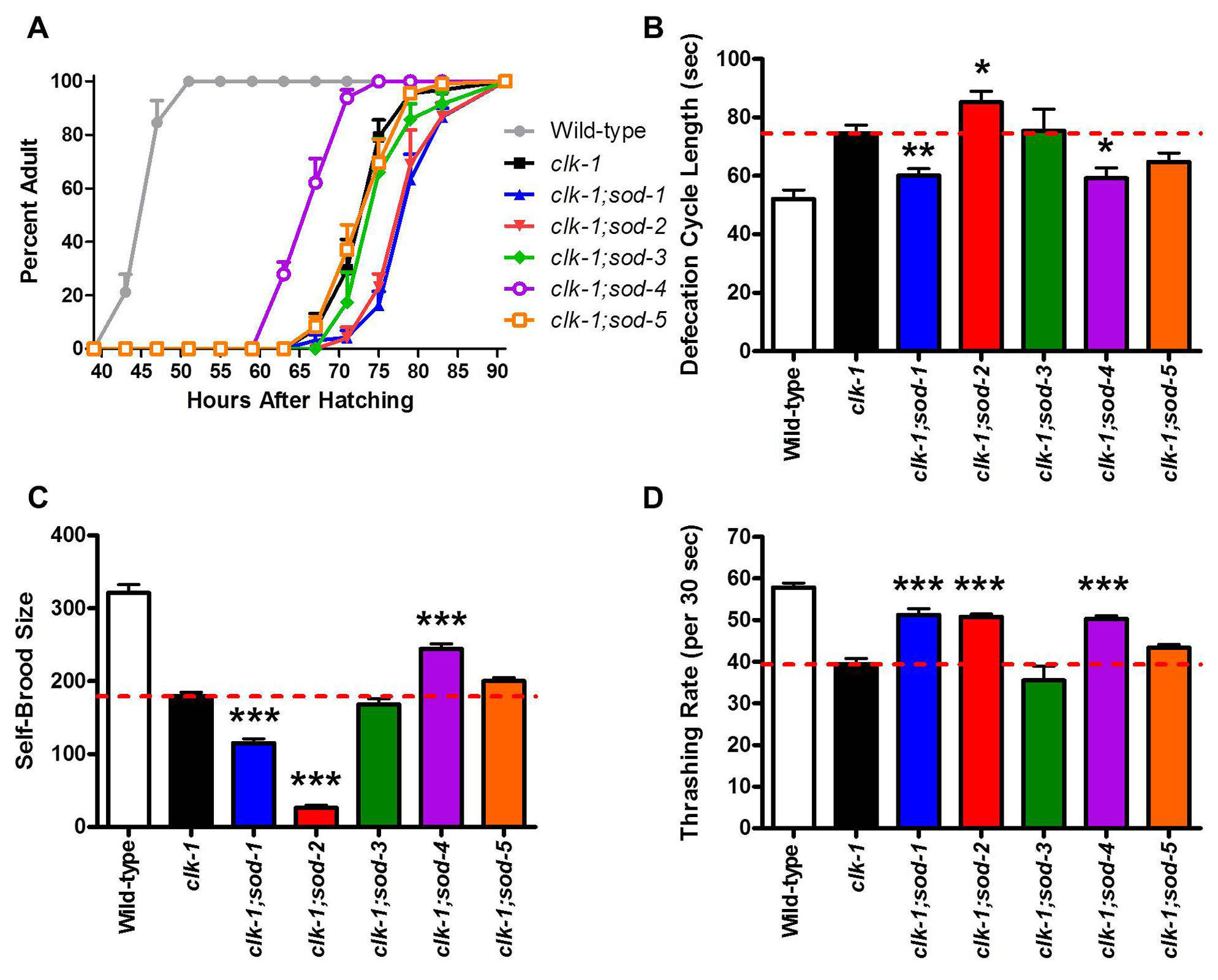

Supplement: S5 Fig — A. clk-1 worms develop slower than wild-type worms. The post-embryonic development time of clk-1 worms was further slowed by deletion of sod-1 or sod-2 but partially rescued by deletion of sod-4. B. The defecation cycle length of clk-1 worms is slower than wild-type worms. This phenotype is exacerbated by deletion of sod-2 and rescued by deletion of sod-1 or sod-4. C. clk-1 worms have decreased brood size compared to wild-type worms. clk-1 brood size is further decreased by deletion of sod-1 or sod-2 but restored towards wild-type by deletion of sod-4. D. The thrashing rate of clk-1 worms is slower than wild-type worms and is restored towards wild-type by deletion of sod-1, sod-2 or sod-4. Significance indicates difference from clk-1 worms. Error bars indicate SEM. *p<0.05, **p<0.01, ***p<0.001. (TIF) [file pgen.1004972.s005.tif]

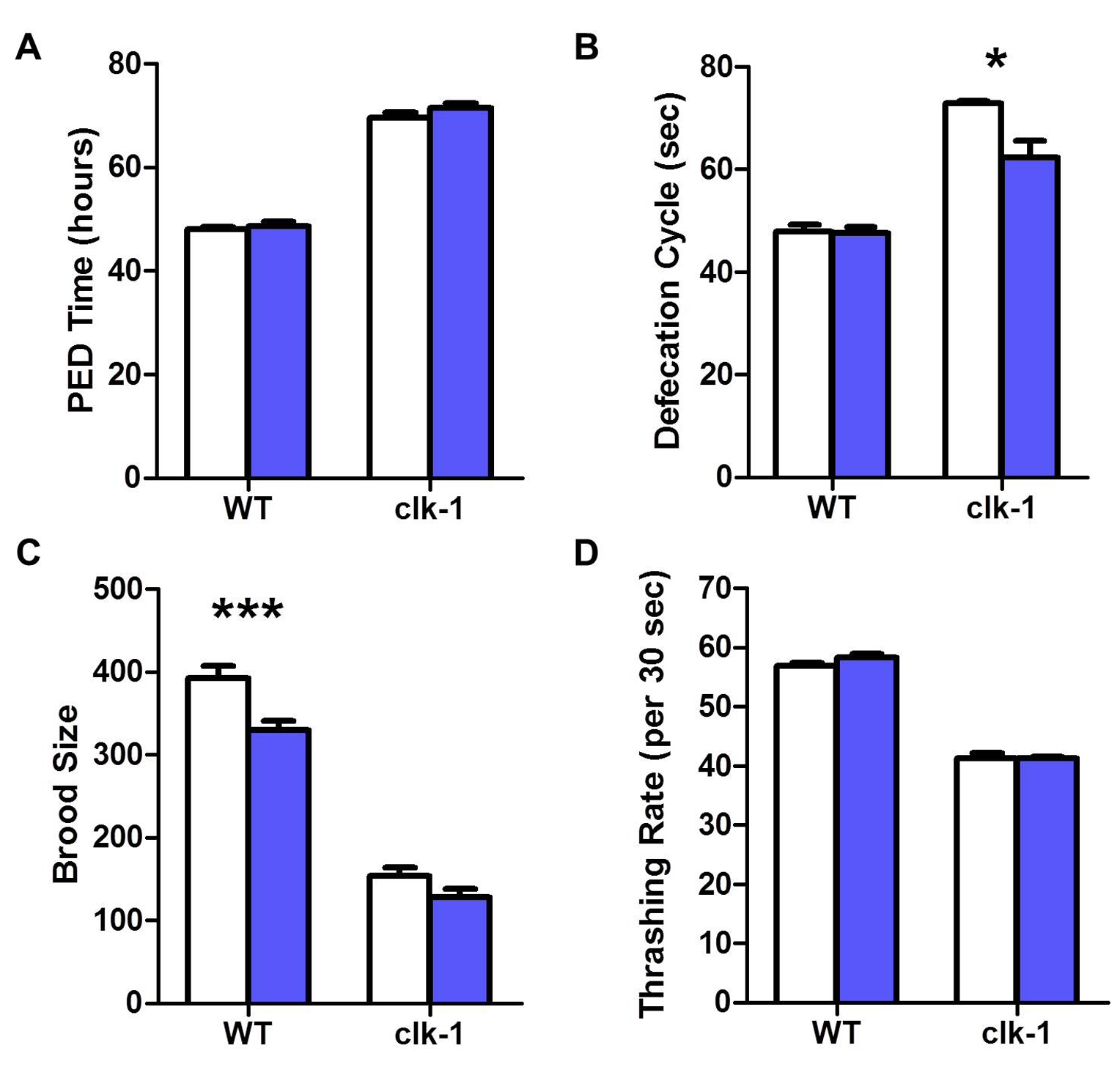

Supplement: S6 Fig — clk-1 worms were treated with 10 mM vitamin C beginning at the L4 stage. Physiologic rates were assessed in the F1 progeny. This concentration of vitamin C was sufficient to decrease clk-1 lifespan A. Treatment with vitamin C did not affect the development time of clk-1 or WT worms. B. Treatment with vitamin C partially rescued the slow defecation cycle length in clk-1 worms but had no effect in WT worms. C. Treatment with vitamin C significantly decreased the brood size of WT worms but not clk-1 worms. D. Thrashing rate was not affected by treatment with vitamin C. Error bars indicate SEM. * p<0.05, *** p<0.001. (TIF) [file pgen.1004972.s006.tif]

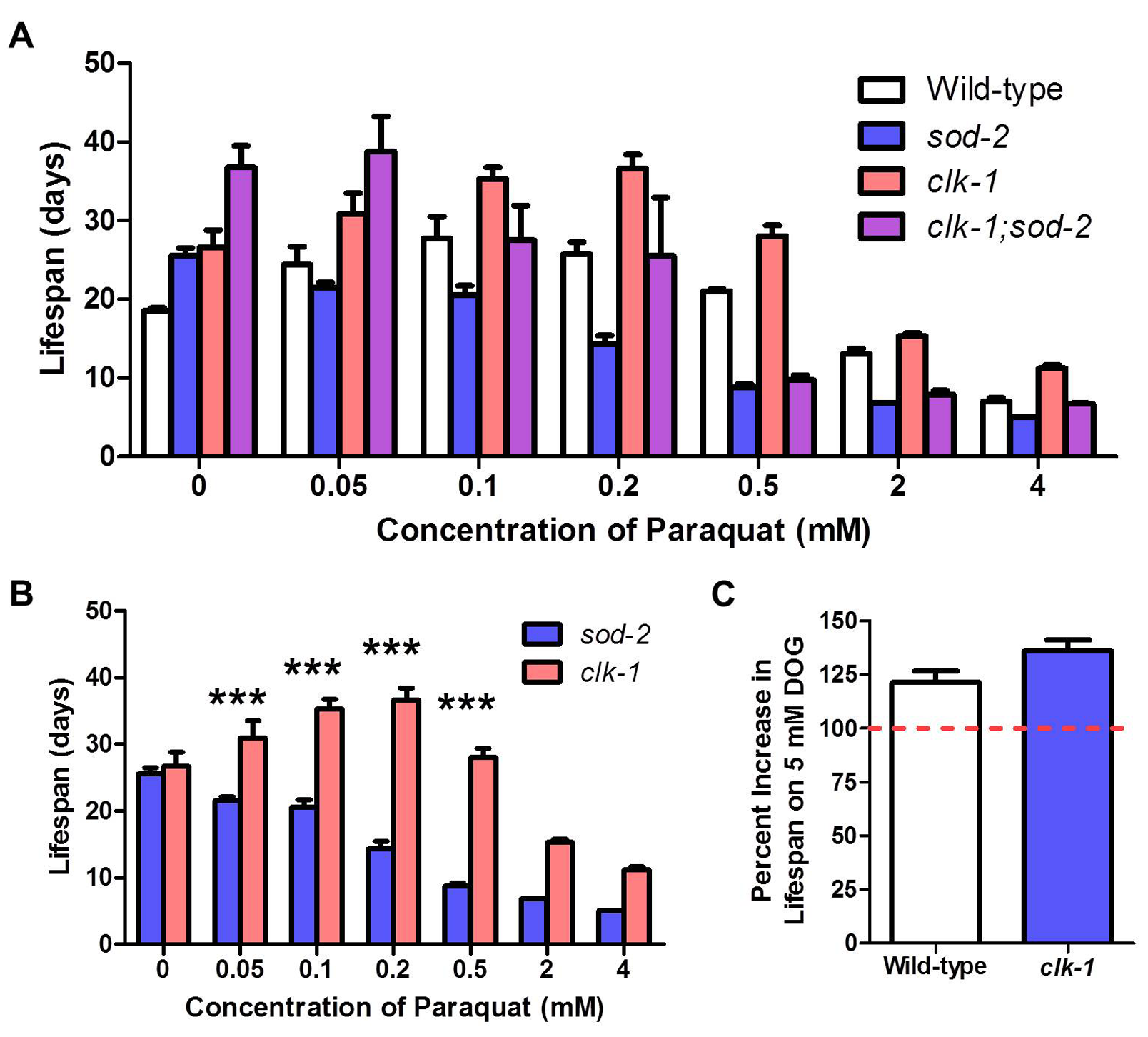

Supplement: S7 Fig — A,B. As in WT worms, treatment of clk-1 worms with low concentrations of paraquat results in increased lifespan. In contrast, treating sod-2 worms with paraquat only results in decreased lifespan. As the longevity of sod-2 worms results from elevated mitochondrial superoxide, this suggests that clk-1 worms also have a ROS-independent mechanism of lifespan extension. Interestingly, the maximum lifespan across the concentration series is similar for clk-1 worms and clk-1;sod-2 worms, as it is for WT and sod-2 worms. In both cases, the optimum concentration of paraquat is decreased by the sod-2 mutation. The presence of the clk-1 mutation increases lifespan above the maximum lifespans achieved by sod-2 deletion or treatment with paraquat alone. C. As with paraquat, increasing ROS through treatment with 2-deoxyglucose (DOG) increases the lifespan of clk-1 and WT worms. Error bars indicate SEM. *** p<0.001. (TIF) [file pgen.1004972.s007.tif]

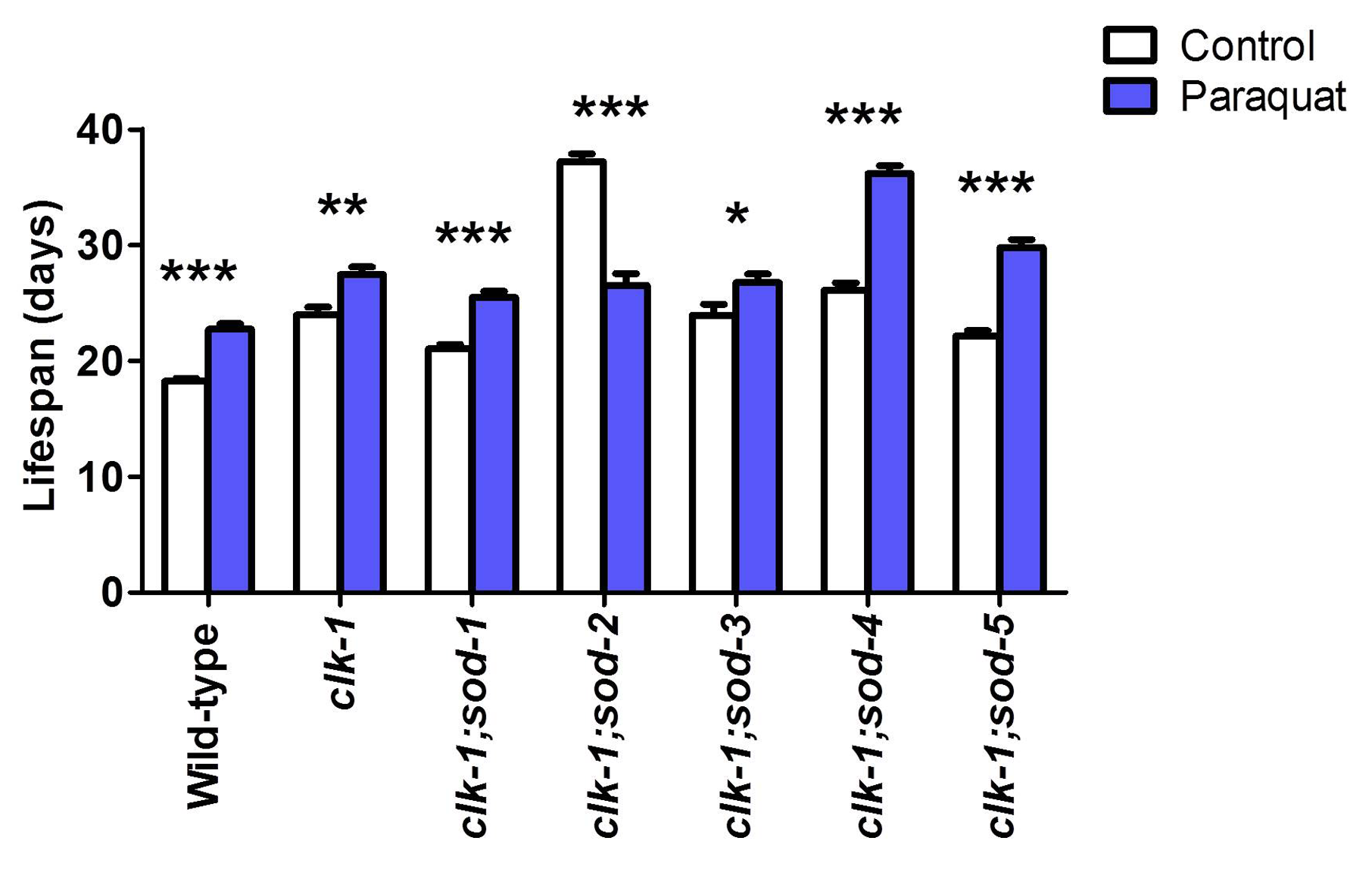

Supplement: S8 Fig — Treatment with low concentrations of paraquat (0.1 mM) is able to extend the lifespan of WT, clk-1, clk-1;sod-1, clk-1;sod-3, clk-1;sod-4 and clk-1;sod-5 double mutants. In contrast, the same treatment resulted in a significant reduction in clk-1;sod-2 lifespan. Error bars indicate SEM. *p<0.05, **p<0.01, ***p<0.0001. (TIF) [file pgen.1004972.s008.tif]

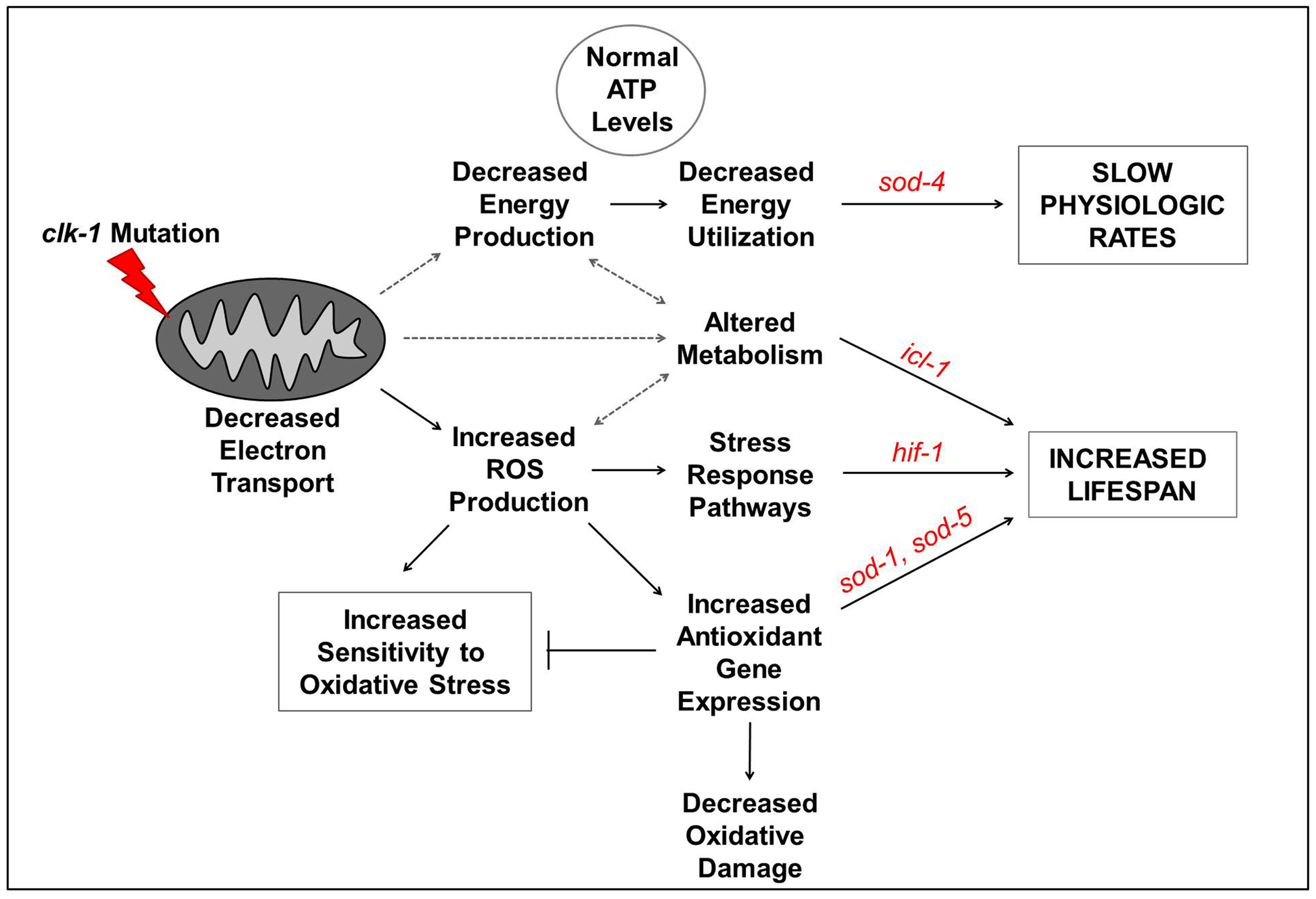

Supplement: S9 Fig — The clk-1 mutation leads to decreased levels of oxidative phosphorylation and increased production of ROS. Elevated ROS cause increased sensitivity to oxidative stress and leads to increased expression of antioxidant genes. The increase in antioxidant gene expression results in increased resistance to chronic oxidative stress and decreased oxidative damage. Elevated ROS in clk-1 worms also activate stress-responsive pathways (e.g. hypoxic response) that contribute to clk-1 longevity. The slow physiologic rates in clk-1 worms can be experimentally dissociated from their longevity and likely result from decreased energy utilization, as clk-1 worms have normal levels of ATP despite decreased energy production. Altered metabolism also contributes to clk-1 longevity. Grey arrows indicate hypothetical connections. Red text indicates genes that are required for the associated arrow. (TIF) [file pgen.1004972.s009.tif]

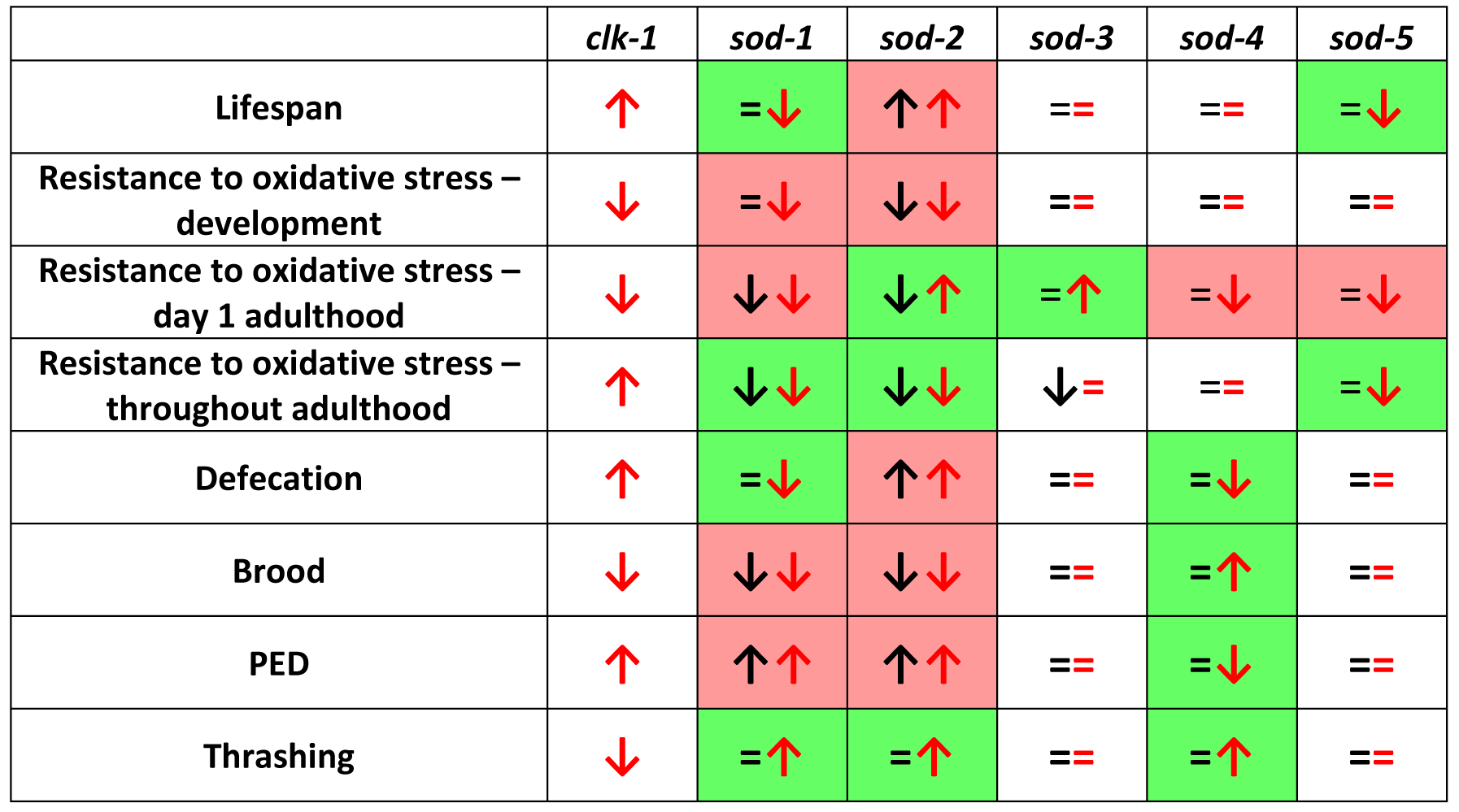

Supplement: S1 Table — Black text indicates the effect of sod gene deletion on wild-type worms. Red text indicates the effect of sod gene deletion in clk-1 worms. Red highlighting indicates sod gene deletion resulting in an exacerbation of the clk-1 phenotype. Green highlighting indicates sod gene deletion resulting in an amelioration of the clk-1 phenotype. Up arrows indicate increase, down arrows indicate decrease, = indicate no change. Date compiled from Figs. 5, 6 and S6. (TIF) [file pgen.1004972.s010.tif]
